# Secretion of Rhoptry and Dense Granule Effector Proteins by Nonreplicating *Toxoplasma gondii* Uracil Auxotrophs Controls the Development of Antitumor Immunity

**DOI:** 10.1371/journal.pgen.1006189

**Published:** 2016-07-22

**Authors:** Barbara A. Fox, Kiah L. Sanders, Leah M. Rommereim, Rebekah B. Guevara, David J. Bzik

**Affiliations:** Department of Microbiology and Immunology, Geisel School of Medicine at Dartmouth, Lebanon, New Hampshire, United States of America; George Washington, University Medical Center, UNITED STATES

## Abstract

Nonreplicating type I uracil auxotrophic mutants of *Toxoplasma gondii* possess a potent ability to activate therapeutic immunity to established solid tumors by reversing immune suppression in the tumor microenvironment. Here we engineered targeted deletions of parasite secreted effector proteins using a genetically tractable Δ*ku80* vaccine strain to show that the secretion of specific rhoptry (ROP) and dense granule (GRA) proteins by uracil auxotrophic mutants of *T*. *gondii* in conjunction with host cell invasion activates antitumor immunity through host responses involving CD8α^+^ dendritic cells, the IL-12/interferon-gamma (IFN-γ) T_H_1 axis, as well as CD4^+^ and CD8^+^ T cells. Deletion of parasitophorous vacuole membrane (PVM) associated proteins ROP5, ROP17, ROP18, ROP35 or ROP38, intravacuolar network associated dense granule proteins GRA2 or GRA12, and GRA24 which traffics past the PVM to the host cell nucleus severely abrogated the antitumor response. In contrast, deletion of other secreted effector molecules such as GRA15, GRA16, or ROP16 that manipulate host cell signaling and transcriptional pathways, or deletion of PVM associated ROP21 or GRA3 molecules did not affect the antitumor activity. Association of ROP18 with the PVM was found to be essential for the development of the antitumor responses. Surprisingly, the ROP18 kinase activity required for resistance to IFN-γ activated host innate immunity related GTPases and virulence was not essential for the antitumor response. These data show that PVM functions of parasite secreted effector molecules, including ROP18, manipulate host cell responses through ROP18 kinase virulence independent mechanisms to activate potent antitumor responses. Our results demonstrate that PVM associated rhoptry effector proteins secreted prior to host cell invasion and dense granule effector proteins localized to the intravacuolar network and host nucleus that are secreted after host cell invasion coordinately control the development of host immune responses that provide effective antitumor immunity against established ovarian cancer.

## Author Summary

*Toxoplasma gondii* extensively manipulates cellular signaling pathways and host immune responses through secreted effector proteins, yet the host rapidly establishes T cell immunity to control acute infection thereby permitting survival of the host as well as survival of the parasite in latent infection. Recently, vaccination of mice bearing highly aggressive ovarian cancer with a safe nonreplicating, noncyst forming, vaccine strain of *T*. *gondii* was shown to effectively reverse tumor associated immune suppression and activate potent antitumor immunity. Using a new genetically tractable Δ*ku80* vaccine strain of *T*. *gondii* we deleted multiple parasite secreted effector molecules to explore parasite specific mechanisms associated with the development of potent antitumor immunity. Our results demonstrate that specialized effector proteins secreted by *T*. *gondii* both before and after host cell invasion trigger and coordinately control the development of a potent antitumor response. Consequently, tracking and understanding the host cell pathways manipulated by these *T*. *gondii* secreted effector proteins can reveal fundamental mechanisms controlling immunity to infection and can also identify relevant mammalian cell mechanisms as new targets for devising more effective therapies against highly aggressive solid tumors.

## Introduction

*Toxoplasma gondii* is a ubiquitous parasite that chronically infects a wide array of warm-blooded vertebrates following the oral ingestion of infectious oocysts or tissue cysts in contaminated water or food [1]. The primary infection is typically subclinical with minor or no apparent disease due to strong immune control, yet *T*. *gondii* invariably establishes long-term infection of the host by developing latent tissue cysts [1]. Infection during pregnancy can harm the fetus, and reactivation of latent stages because of immune deficiency (AIDS, cancer chemotherapy, transplantation) causes severe and potentially lethal toxoplasmosis infections [2]. There are no currently approved vaccines to prevent toxoplasmosis in humans, or vaccines to prevent infection of cats which host the sexual parasite stages and disseminate infectious oocysts into the environment [3]. Remarkably, uracil auxotrophic vaccine strains of *T*. *gondii* that do not replicate or cause infection in mammals retain a dynamic ability to activate protective immunity to *T*. *gondii* [4–14] as well as protective immunity to established highly aggressive pancreatic, melanoma, and ovarian tumors [15–20].

The remarkable biological ability of *T*. *gondii* to manipulate the immune system most likely originates from its life style as an obligate intracellular parasite. The parasite as well as the host must both survive the acute infection to permit the development of latent infection that is essential for the transmission of *T*. *gondii* to new hosts [21]. To accomplish this, *T*. *gondii* extensively manipulates its host cells through the secretion of specialized effector proteins [21,22]. Secreted rhoptry (ROP) effector proteins originating from the apical rhoptry organelle are injected directly into the host cell cytosol prior to active invasion of the host cell and formation of the parasitophorous vacuole (PV) [23,24]. After host cell invasion, many of these ROP effectors traffic specifically to the nascent PV membrane (PVM) to establish PVM functions required for parasite replication and survival [23,25]. Rhoptry secreted effectors are also injected into parasite contacted host cells that are not subsequently invaded [26–28], suggesting that parasite manipulation of host cells occurs in both the parasite invaded as well as in the parasite injected noninvaded cell populations. After PVM formation, effectors are secreted from parasite dense granules (GRA proteins) into the PV lumen and these GRA proteins traffic to the intravacuolar network (IVN) of nanotubular membranes, to the PVM and its extensions, to the host cell cytosol, or to the host cell nucleus [29]. The PV avoids acidification and fusion with host endolysosomes [30,31], and host cell endoplasmic reticulum (ER) and mitochondria closely associate with the PVM [30,32,33]. A parasite secreted protein (MAF1) mediates the association of mitochondria to the PVM and modulates host inflammatory cytokines [34].

The host rapidly recognizes *T*. *gondii* and drives a T_H_1-biased immune response marked by the cytokine IL-12 [35]. CD8α^+^ dendritic cells drive the initial IL-12 response downstream of TLR11/12 mediated activation of MyD88 [36–38]. Production of IL-12 is required for the development of protective T cell populations [11,12] and immune control is ultimately achieved by T cell populations that produce the cytokine interferon-gamma (IFN-γ) [7,8,11,39,40], and by cytolytic CD8^+^ T cell populations [8,41]. IFN-γ activates cell autonomous host immunity related GTPases (IRGs) [42–47]. In addition, host guanylate binding proteins associate with the PVM [48–53] and components of autophagy also associate with the PVM after exposure to IFN-γ but fail to activate degradative macroautophagy [54–58].

*T*. *gondii* strain types exhibit variation in their level of virulence. Virulent type I strains invariably kill laboratory and outbred strains of mice, while type II and III strains are markedly less virulent [3]. Strain type dependent virulence is linked to polymorphisms in secreted proteins such as ROP5 and ROP18 that form PVM associated complexes to resist IFN-γ activated IRG dependent innate killing mechanisms [59–72], and virulence is also correlated with the exact ROP5 and ROP18 allele combinations [73]. ROP5 and ROP18 were also recently identified as virulence factors in divergent South American strains of *T*. *gondii* [74] and in less virulent type II strains [75]. The ROP18 kinase activity directly inactivates innate immunity by phosphorylation of host IRGs [63]. ROP18 also inhibits host transcription factor NF-κβ signaling through association with its p65 subunit [76] and ROP18's association with the host ER stress sensor ATF6β interferes with antigen presentation [77]. Thus ROP18 manipulates innate and adaptive immunity as well as host cell signaling pathways. The ROP38 secreted effector molecule extensively modulates host cell transcriptional programming and gene expression profiles [78], and while this molecule does not influence parasite virulence, it is required for establishing latent infection [75]. Dense granule (GRA) secreted effectors also play important roles in host cell manipulation [34,79–82]. In particular, ROP16 [83–85], GRA15 [86], and GRA24 [87] manipulate host cell signaling pathways and their associated biological outputs can either promote or inhibit production of IL-12 by the invaded host cell in a parasite strain type dependent manner. Parasite modulation of the IL-12 response is significant because *T*. *gondii* preferentially invades dendritic cells [88] that are responsible for initiating the host IL-12 response necessary for the development of protective T cell populations [89]. Consequently, active invasion of the host cell by *T*. *gondii* uniquely creates a nonfusogenic PV hospitable for intracellular parasite replication and parasite infection while extensively modifying host cell behavior through functions of ROP and GRA secreted effector proteins [21,22].

Uracil auxotrophic vaccine strains [5,13,14] have emerged as important models to dissect the parasite-host interaction and to investigate host and parasite mechanisms that determine the development of life-long CD8^+^ T cell dependent immunity [4–14]. Nonreplicating uracil auxotrophs permit the evaluation of infected host cells manipulated by secreted effector proteins in the absence of parasite replication and destruction of the host cell. Uracil auxotrophs preferentially invade myeloid cell types [4,16–19] and more quickly stimulate protective CD8^+^ T cell responses in comparison to replicating strains of *T*. *gondii* [4,7,8,14,18]. Nonreplicating uracil auxotrophs also preferentially target and invade CD11c^+^ and myeloid cell populations in aggressive models of murine pancreatic cancer, melanoma, and ovarian cancer to convert tolerogenic tumor microenvironments into immune stimulatory microenvironments [15,16,19,20]. Uracil auxotrophs stimulate high-level expression of co-stimulatory molecules CD80 and CD86 as well as IL-12, and tumor antigen specific CD8^+^ T cell populations that mediate the long-term survival of tumor-bearing mice [15,16,19,20].

Here, we developed targeted deletion mutants using genetically tractable Δ*ku80* uracil auxotrophic vaccine strains to explore the extent to which parasite secreted ROP and GRA effector proteins are associated with mechanisms that promote CD8^+^ T cell dependent therapeutic immunity to established highly aggressive ovarian tumors [16,90]. Our results reveal that rhoptry secretion, host cell invasion, formation of the PVM, formation of the IVN, and the secretion of dense granule proteins to the host cell nucleus are essential for the development of the antitumor response. The ROP18 kinase activity that mediates parasite virulence was not essential for this antitumor response. However, ROP18 PVM localization, other PVM localized rhoptry molecules (ROP5, ROP17, ROP35 and ROP38), and dense granule molecules that localized to the IVN (GRA2 and GRA12) or to the host cell nucleus (GRA24) were essential to trigger the development of this potent antitumor immunity.

## Results

### Immunity to *T*. *gondii* does not diminish the potency of the antitumor response stimulated by uracil auxotrophs

**Fig 1:**
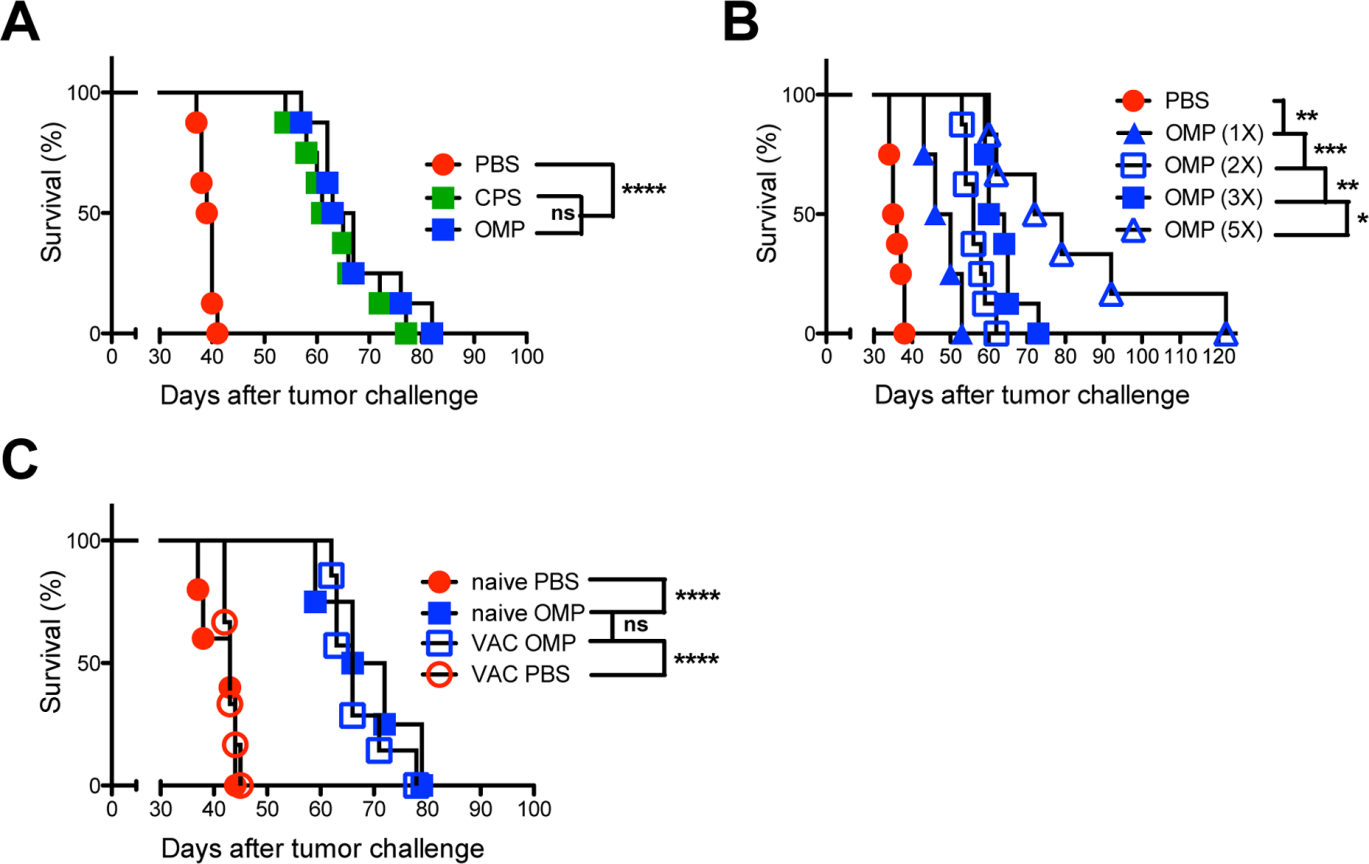
Immunity to *T*. *gondii* does not diminish the potency of the antitumor response stimulated by uracil auxotrophs. (A) ID8DV ovarian tumors were established in C57BL/6 mice and groups of mice were treated with phosphate buffered saline (PBS) or mice were vaccinated i.p. with tachyzoites (the acute replicative form of *T*. *gondii*) of uracil auxotrophs (OMP [13] or CPS [5]) at 8, 20, and 32 d after tumor challenge (the three-dose treatment schedule). (B) ID8DV ovarian tumors were established in C57BL/6 mice and groups of mice were treated with PBS or were vaccinated once (8 d), twice (8, 20 d), three times (8, 20, 32 d), or five times (8, 20, 32, 44, 56 d) i.p. with tachyzoites of uracil auxotrophs. (C) Groups of C57BL/6 mice were vaccinated (VAC) with uracil auxotrophs to establish protective immunity or mice were treated with PBS (naive). Twelve months later ID8DV tumors were established in vaccinated or age matched naive mice and tumors were treated with PBS or were vaccinated with uracil auxotrophs using the three-dose treatment schedule. Data is representative of at least two independent experiments. ns was not significant, *p<0.05, **p<0.01, ***P<0.001, ****p<0.0001.

Therapeutic vaccination of established highly aggressive and immunosuppressive ID8-*Defβ29*/*Vegf-A* (ID8DV) ovarian tumors [90,91] with the nonreplicating type I uracil auxotrophic vaccine strain CPS [5] was shown to preferentially target CD11c^+^ antigen presenting cells to reverse tumor associated immune suppression and promote tumor antigen specific CD8^+^ T cell dependent antitumor immunity [16–18]. To identify parasite and host mechanisms that mediate this potent antitumor response we utilized a genetically tractable nonreverting CPS-like type I uracil auxotrophic vaccine strain (the OMP mutant) [13] developed in the RHΔ*ku80* background [92] that enables highly efficient targeted genetic manipulations. We first verified that a three-dose OMP or CPS intraperitoneal vaccination protocol delivered sequentially at 8, 20, and 32 days after ID8DV ovarian tumor challenge markedly prolonged the survival of tumor-bearing mice to a similar degree (Fig 1A). OMP vaccination delivered systemically by the intravenous route provided a mean survival of 54 days while intraperitoneal vaccination provided a mean survival of 59 days (S1A Fig). Additional OMP vaccinations delivered at 12-day intervals markedly enhanced the survival of ovarian tumor-bearing mice (Fig 1B), suggesting that the underlying therapeutic immune responses were triggered even after immunity to *T*. *gondii* was established. Moreover, aged mice with established long-term vaccine induced immunity to *T*. *gondii* infection (S1B Fig) exhibited equivalent therapeutic benefits to those of age matched nonimmune naive mice (Fig 1C). These results suggest that previous protective immune responses induced by uracil auxotrophs in tumor-bearing mice did not abrogate the efficacy of additional sequentially delivered vaccinations with uracil auxotrophs.

### The IL-12/IFN-γ T_H_1 axis is required for optimal antitumor responses

**Fig 2:**
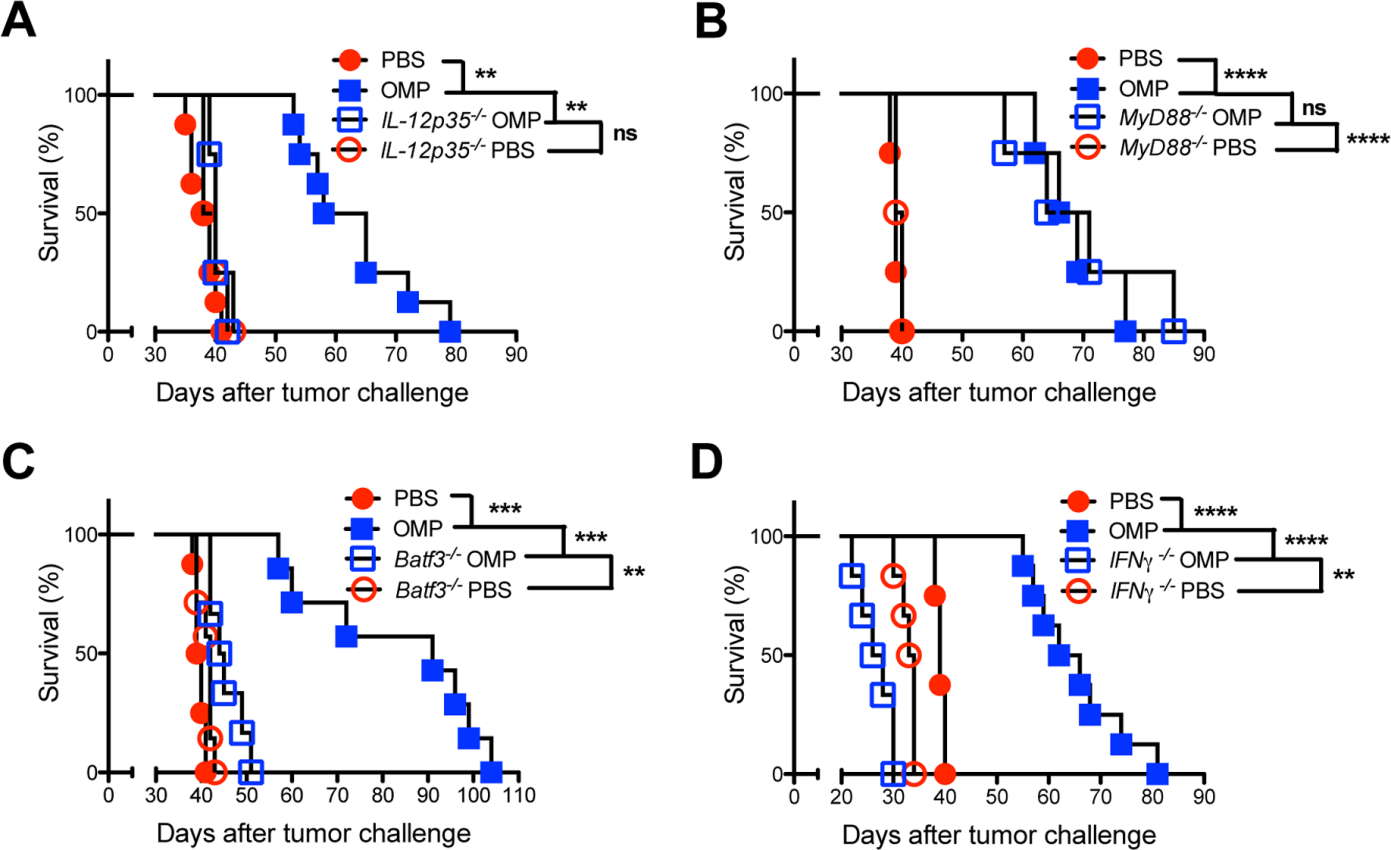
IL-12, CD8α^+^ dendritic cells, and IFN-γ are essential for stimulation of the antitumor response elicited by uracil auxotrophs. ID8DV ovarian tumors were established and groups of mice were treated with PBS or were vaccinated i.p. with tachyzoites of uracil auxotrophs (OMP) using the three-dose treatment schedule. (A) Wild-type or *IL-12p35*^*-/-*^ C57BL/6 mice. (B) Wild-type or *MyD88*^*-/-*^ C57BL/6 mice. (C) Wild-type or *Batf3*^*-/-*^ C57BL/6 mice. (D) Wild-type or *IFN-*γ^*-/-*^ C57BL/6 mice. Data is representative of two independent experiments. ns was not significant, **p<0.01, ***P<0.001, ****p<0.0001.

IL-12 was previously shown to be important to the antitumor response to ID8DV tumors induced by therapeutic vaccination with uracil auxotrophs [16]. To verify the involvement of the host IL-12/IFN-γ T_H_1 axis in the antitumor response we vaccinated ID8DV tumor-bearing *IL-12p35*^*-/-*^ mice with uracil auxotrophs. *IL-12p35*^*-/-*^ mice lacking the ability to produce bioactive IL-12p70 (IL12-p35 + IL12-p40 subunits) mounted no detectable antitumor response (Fig 2A). *IL-12p40*^*-/-*^ mice also exhibited no antitumor response after vaccination (S2 Fig). Despite the recognized importance of MyD88 signaling in driving high level IL-12 production in response to *T*. *gondii* infection [37], vaccination of *MyD88*^*-/-*^ mice bearing ID8DV tumors provided a therapeutic benefit equivalent to vaccination of wild-type tumor-bearing mice (Fig 2B). Together these results suggested that IL-12 production via a MyD88 independent mechanism was sufficient to drive the antitumor response induced by uracil auxotrophs.

The CD8α^+^ dendritic cell subset is a critical source of IL-12 required for immune control of *T*. *gondii* infection [36]. To address the role of CD8α^+^ dendritic cells in the antitumor response we established ID8DV tumors in basic leucine zipper transcription factor ATF-like 3 deficient (*Batf3*^*-/-*^) mice which exhibit a loss of CD8α^+^ dendritic cells [93], and vaccinated these tumors. The antitumor response triggered by vaccination with uracil auxotrophs was severely abrogated in *Batf3*^*-/-*^ mice (Fig 2C). These data argue that the CD8α^+^ dendritic cell subset confers critical mechanisms required for the stimulation of antitumor immunity.

Production of protective IFN-γ in response to *T*. *gondii* infection is highly dependent on production of IL-12 [35]. To investigate the role for IFN-γ in the antitumor response we established ID8DV tumors in IFN-γ deficient mice (*IFN-*γ^*-/-*^) and vaccinated these mice. Production of IFN-γ was required for the therapeutic benefit (Fig 2D). In addition, IFN-γ deficient mice developed ID8DV ovarian tumors more rapidly and succumbed faster to ovarian cancer than wild-type mice (Fig 2D). Collectively, these results suggested that CD8α^+^ dendritic cells play a key role in stimulating the IL-12/IFN-γ T_H_1 axis to promote antitumor immunity.

### CD4^+^ and CD8^+^ T cells are required for optimal antitumor responses

**Fig 3:**
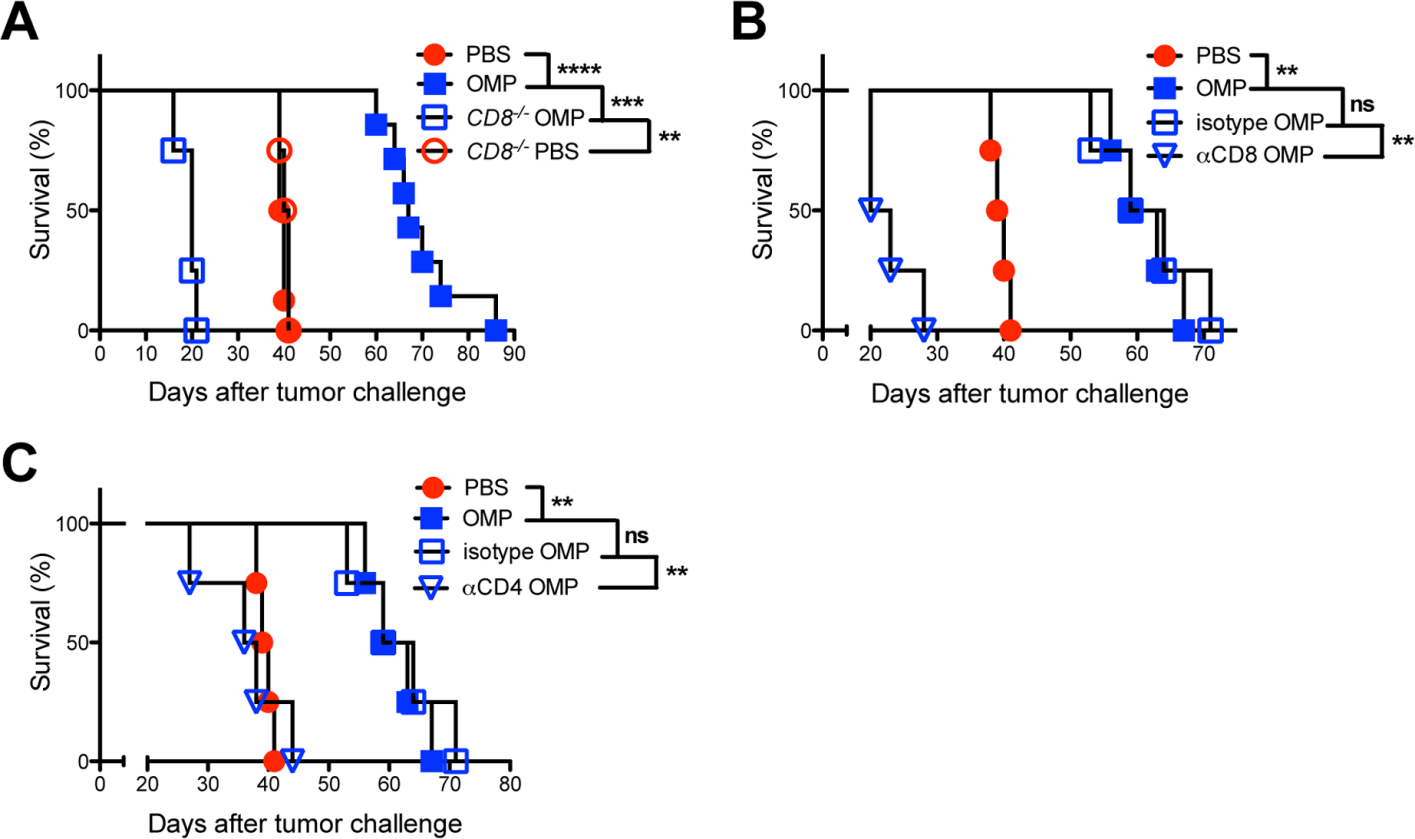
CD8^+^ and CD4^+^ T cells are required for the antitumor response. (A) ID8DV ovarian tumors were established in wild-type or *CD8*^*-/-*^ C57BL/6 mice and groups of mice were treated with PBS or were vaccinated i.p. with tachyzoites of uracil auxotrophs (OMP) using the three-dose treatment schedule. (B-C) ID8DV ovarian tumors were established in C57BL/6 mice and groups of mice were treated with PBS or were vaccinated i.p. with tachyzoites of uracil auxotrophs (OMP) on day 8 and day 20. Groups of mice were treated with isotype control antibody, or (B) αCD8 antibody, or (C) αCD4 antibody on days 7, 8, 11, 19, 20, 23 after tumor challenge. Data is representative of two independent experiments. ns was not significant, **p<0.01, ***P<0.001, ****p<0.0001.

We previously reported that therapeutic vaccination of established ID8DV ovarian tumors with uracil auxotrophs stimulated a significant increase in ovarian tumor antigen specific CD8^+^ T cells as well as total T cell populations that limited ovarian tumor development [16]. To verify the protective T cell populations required for this antitumor immunity we examined tumor development in CD8 deficient mice (*CD8*^*-/-*^). Vaccinated tumor-bearing *CD8*^*-/-*^ mice exhibited no therapeutic benefit, and treatment accelerated disease in *CD8*^*-/-*^ mice (Fig 3A). Similar results were observed after depletion of CD8^+^ T cells using anti-CD8α antibody (Fig 3B). NK cells provide another early pathway of IFN-γ production in response to *T*. *gondii* infection [94]. However, depletion of this cell population using anti-NK1.1 antibody did not affect the antitumor response (S3A Fig).

Depletion of CD4^+^ T cells using anti-CD4 antibody also abrogated the antitumor response (Fig 3C). Moreover, consistent with a requirement for CD4^+^ T cells, major histocompatibility complex (MHC) II was also required for stimulation of the antitumor response (S3B Fig). Surprisingly, MHCII played a dichotomous role. The absence of MHCII in normal (nontumor) cells delayed development of the ID8DV tumor, suggesting that MHCII may also act to promote ovarian tumor development (S3B Fig). Together these results suggested that CD8^+^ T cells, CD4^+^ T cells, and MHCII were essential to drive IFN-γ production and antitumor immunity.

### Live invasive parasites are required for stimulation of the IL-12/IFN-γ T_H_1 axis

**Fig 4:**
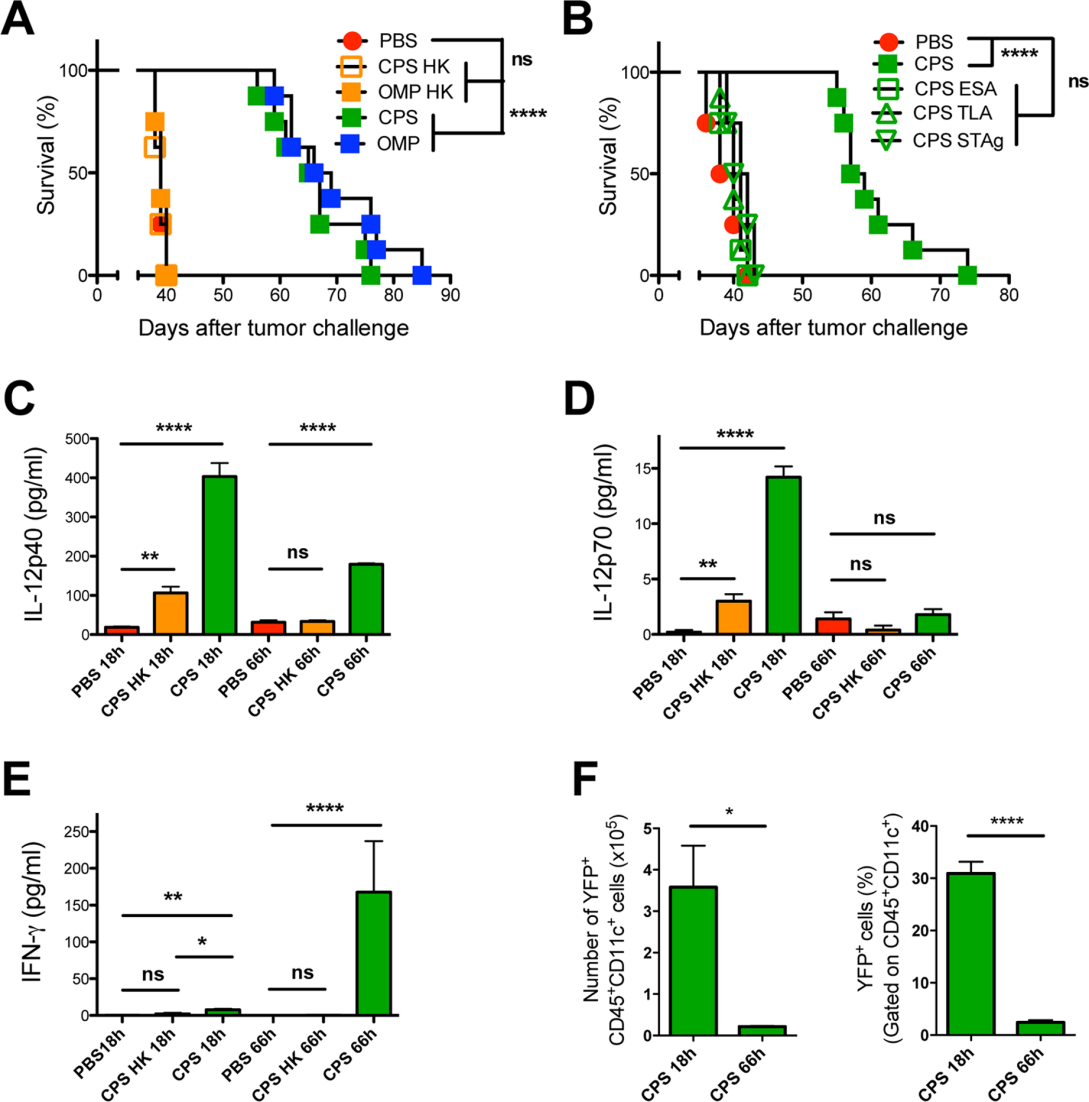
Parasite extracts and heat killed uracil auxotrophs fail to stimulate antitumor responses or IFN-γ production in the tumor microenvironment. (A) ID8DV ovarian tumors were established in C57BL/6 mice and groups of mice were treated with PBS, vaccinated i.p. with tachyzoites of uracil auxotrophs (OMP or CPS) or vaccinated i.p. with heat killed (HK) tachyzoites of uracil auxotrophs (OMP or CPS) using the three-dose treatment schedule. (B) ID8DV ovarian tumors were established in C57BL/6 mice and groups of mice were treated with PBS, vaccinated i.p. with tachyzoites of uracil auxotrophs (CPS) or vaccinated i.p. with excreted/secreted protein (ESA) extracts, tachyzoite lysate antigen (TLA) extracts, or soluble tachyzoite antigen (STAg) extracts using the three-dose treatment schedule. (C-E) ID8DV tumors were established in groups of C57BL/6 mice for 25 days then tumor-bearing mice were treated with PBS, vaccinated with heat killed (HK) uracil auxotrophs or vaccinated with live uracil auxotrophs (experiments used the yellow fluorescent protein expressing CPS strain CPS-YFP [96]) and peritoneal levels of (C) IL-12p40, (D) IL-12p70, (E) IFN-γ, and (F) the absolute number or percentage of YFP^+^CD11c^+^ cells present in the tumor microenvironment were measured 18 and 66 h after treatments. Data is representative of two independent experiments. ns was not significant, *p<0.05, **p<0.01, ****P<0.0001.

To examine whether active invasion of host cells was essential to the antitumor response, we heat killed uracil auxotrophs at 56°C to abolish their ability to invade host cells (S4A Fig). Heat killed uracil auxotrophs exhibited no detectable antitumor response (Fig 4A). However, we could not exclude the possibility that heat treatment reduced the activity of parasite molecules that would otherwise stimulate IL-12 production, since heat treatment of *T*. *gondii* has been previously shown to elicit a reduced IL-12 response in comparison to live parasites [95]. To address this, we prepared, tachyzoite excreted/secreted antigen (ESA) extracts, tachyzoite lysate antigen (TLA) extracts, and soluble tachyzoite antigen (STAg) extracts from uracil auxotrophs to examine whether parasite extracts not subjected to heat treatment could promote the antitumor response. ESA, TLA, and STAg extracts exhibited no detectable antitumor activity (Fig 4B).

We hypothesized that while heat killed uracil auxotrophs could stimulate IL-12, the activation of the IL-12/IFN-γ T_H_1 axis may be incomplete. To examine this, highly immunosuppressive ID8DV tumors were established in mice for 25 days and vaccinated once with live uracil auxotrophs or heat killed uracil auxotrophs. Supernatants from the peritoneal ovarian tumor microenvironment were analyzed for IL-12, type I and type II interferons, and inflammasome activation 18 and 66 h post-vaccination. Increased production of IL-12p40 and IL-12p70 was observed 18 h following treatment with heat killed parasites but this early IL-12 subsided by 66 h (Fig 4C and 4D). As expected, higher levels of IL-12 were observed 18 h after vaccination with live uracil auxotrophs, and this response persisted for at least 66 h (Fig 4C and 4D).

Live invasive uracil auxotrophs elicited a slight increase in IFN-γ as early as 18 h, however, IFN-γ levels were markedly increased by 66 h (Fig 4E). In contrast, noninvasive heat killed parasites failed to stimulate any detectable increase in the production of IFN-γ in the immunosuppressive ID8DV tumor microenvironment (Fig 4E). In addition, type I interferon alpha (IFN-α) was not detected and low levels of interferon beta (IFN-β) present in the untreated tumor microenvironment were not affected by vaccination with either live or with heat killed parasites (S4B Fig). While IL-1β was not detected in the tumor microenvironment at 18 or 66 h post-vaccination, a low level increase in IL-1α was observed at 66 h after therapeutic vaccination with live invasive uracil auxotrophs (S4C Fig).

CD4^+^ and CD8^+^ T cells are the major producers of IFN-γ in *T*. *gondii* infection [39], and IFN-γ is the key inflammatory cytokine that activates the major mechanism of IRG-dependent innate immunity that effectively clears intracellular parasites and PVs [63]. Preferential invasion of CD11c^+^ antigen presenting cells by uracil auxotrophs was previously associated with the initiation of the T cell dependent antitumor response that targets ID8DV ovarian tumors [16]. The requirement of live invasive parasites to induce IFN-γ (Fig 4E) suggested that persistence of parasites in invaded host cells could be essential to drive the antitumor response. On the other hand, IFN-γ could also rapidly clear parasites from invaded cells through host IRGs and innate immunity. Type I uracil auxotrophs were recently shown to fully resist PV clearance in IFN-γ activated macrophages *in vitro* for at least 5 days [4]. We examined clearance of YFP^+^ uracil auxotrophs from invaded CD11c^+^ cells in the ID8DV tumor microenvironment and observed a 16.8-fold and 12.9-fold reduction in the absolute number or percentage, respectively, of YFP^+^CD11c^+^ antigen presenting cells between 18 h and 66 h after therapeutic vaccination (Fig 4F). In addition, previous studies have shown that uracil auxotrophs remain in the peritoneum after vaccination and parasite invaded myeloid cells were found to rarely migrate to draining lymph nodes in either naive mice [4] or in tumor-bearing mice [19]. These results suggest that uracil auxotroph PVs in CD11c^+^ cells were not cleared prior to 18 h but were rapidly cleared between 18 and 66 h, potentially through an IFN-γ dependent mechanism.

### Active invasion and injection of parasite secreted molecules into host cells trigger potent antitumor responses

**Fig 5:**
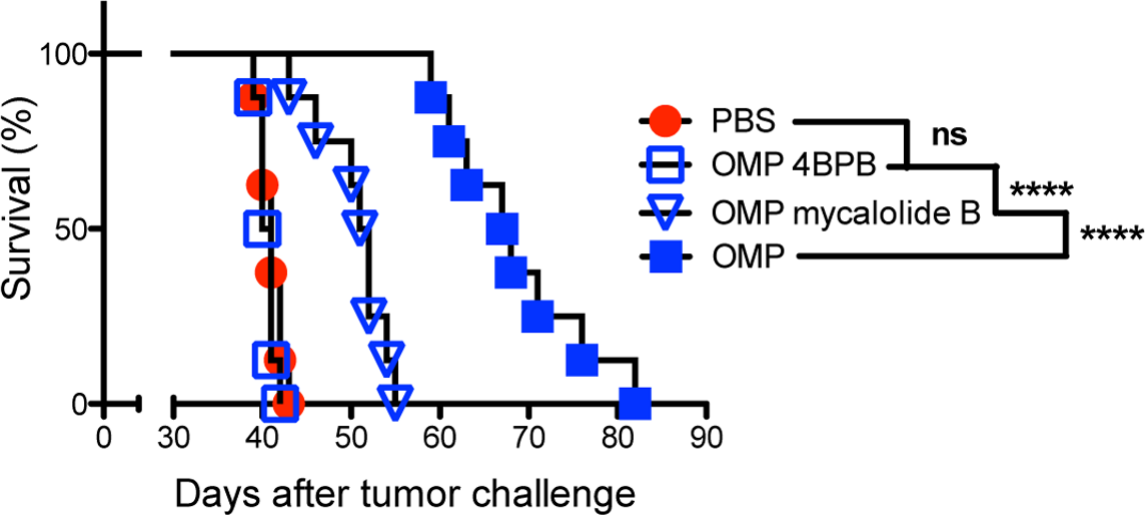
Secretion and active invasion is required for the antitumor response. ID8DV ovarian tumors were established in C57BL/6 mice and groups of mice were treated with PBS, or were vaccinated i.p. with 4BPB treated, mycalolide B treated, or untreated tachyzoites of uracil auxotrophs (OMP) using the standard three-dose treatment schedule. Data is representative of three independent experiments. ns was not significant, ****P<0.0001.

In view that live invasive uracil auxotrophs were associated with the potent antitumor response, we explored whether invasion and/or rhoptry secretion was required for this response. To investigate rhoptry secretion, uracil auxotrophs were treated with the chemical 4-bromophenacyl bromide (4BPB). 4BPB selectively inhibits phospholipase A_2_ to irreversibly block rhoptry secretion and parasite invasion of host cells (S5 Fig) without affecting parasite attachment to host cells [97–99]. 4BPB treatment of uracil auxotrophs completely abrogated the antitumor response (Fig 5), suggesting that rhoptry secretion was essential for this response.

*Toxoplasma* directly injects rhoptry effector molecules into host cells prior to invasion as well as into contacted but noninvaded bystander cells [26–28]. To differentiate the role of rhoptry secretion into host cells with or without parasite invasion and PVM formation in the host cell, we treated uracil auxotrophs with the chemical mycalolide B, a compound that irreversibly blocks parasite motility thereby inhibiting invasion of host cells without blocking rhoptry secretion [100,101]. As expected, mycalolide B treated uracil auxotrophs were blocked in their invasion of host cells (S5 Fig). Mycalolide B treated uracil auxotrophs were markedly weakened in their ability to trigger the antitumor response (Fig 5). Together these chemical inhibition experiments suggested that rhoptry secretion as well as active invasion of host cells was necessary to arm the full antitumor response.

### Parasite secreted molecules derived from rhoptry and dense granules trigger the antitumor response

Based on the requirements for active invasion and parasite secretion, we hypothesized that specific ROP and GRA secreted effector proteins may control the antitumor response. To identify whether specific secreted effector molecules were involved in the antitumor response we used a reverse genetic approach to target complete gene deletions of selected candidate parasite ROP or GRA genes using the Δ*ku80ΔompdcΔupΔhxgprt* OMP uracil auxotroph vaccine background as the parental strain (Table 1). Targeted gene deletions were engineered using positive genetic selection to replace genes of interest (GOI) with the *HXGPRT* selectable marker [102] at the targeted gene loci (S6 Fig) [13,14,92,103,104].

**Table 1:** Genotype of parasite strains developed in this study.

Strain	Parent	Source
RHΔ*ku80Δhxgprt*	RHΔ*ku80*::*HXGPRT*	[92]
RHΔ*ku80ΔompdcΔup*::*HXGPRT*	RHΔ*ku80ΔhxgprtΔompdc*	[13]
RHΔ*ku80ΔompdcΔupΔhxgprt*	RHΔ*ku80ΔompdcΔup*::*HXGPRT*	This paper
RHΔ*ku80ΔompdcΔupΔgra2*::*HXGPRT*	RHΔ*ku80ΔompdcΔupΔhxgprt*	This paper
RHΔ*ku80ΔompdcΔupΔgra3*::*HXGPRT*	RHΔ*ku80ΔompdcΔupΔhxgprt*	This paper
RHΔ*ku80ΔompdcΔupΔgra12*::*HXGPRT*	RHΔ*ku80ΔompdcΔupΔhxgprt*	This paper
RHΔ*ku80ΔompdcΔupΔgra15*::*HXGPRT*	RHΔ*ku80ΔompdcΔupΔhxgprt*	This paper
RHΔ*ku80ΔompdcΔupΔgra16*::*HXGPRT*	RHΔ*ku80ΔompdcΔupΔhxgprt*	This paper
RHΔ*ku80ΔompdcΔupΔgra24*::*HXGPRT*	RHΔ*ku80ΔompdcΔupΔhxgprt*	This paper
RHΔ*ku80ΔompdcΔupΔrop5*::*HXGPRT*	RHΔ*ku80ΔompdcΔupΔhxgprt*	This paper
RHΔ*ku80ΔompdcΔupΔrop16*::*HXGPRT*	RHΔ*ku80ΔompdcΔupΔhxgprt*	This paper
RHΔ*ku80ΔompdcΔupΔrop16Δhxgprt*	RHΔ*ku80ΔompdcΔupΔrop16*::*HXGPRT*	This paper
RHΔ*ku80ΔompdcΔupΔrop16Δgra15*::*HXGPRT*	RHΔ*ku80ΔompdcΔupΔrop16Δhxgprt*	This paper
RHΔ*ku80ΔompdcΔupΔrop17*::*HXGPRT*	RHΔ*ku80ΔompdcΔupΔhxgprt*	This paper
RHΔ*ku80ΔompdcΔupΔrop18*::*HXGPRT*	RHΔ*ku80ΔompdcΔupΔhxgprt*	This paper
RHΔ*ku80Δompdc*::*ROP18ΔupΔrop18*::*HXGPRT*	RHΔ*ku80ΔompdcΔupΔrop18*::*HXGPRT*	This paper
RHΔ*ku80Δompdc*::*ROP18*^*PAH2(ATF)*^*Δ upΔrop18*::*HXGPRT*	RHΔ*ku80ΔompdcΔupΔrop18*::*HXGPRT*	This paper
RHΔ*ku80Δompdc*::*ROP18*^*PAH2(ATF)*,*KD*^*ΔupΔrop18*::*HXGPRT*	RHΔ*ku80ΔompdcΔupΔrop18*::*HXGPRT*	This paper
RHΔ*ku80Δompdc*::*ROP18*^*KD*^*ΔupΔrop18*::*HXGPRT*	RHΔ*ku80ΔompdcΔup*Δ*rop18*::*HXGPRT*	This paper
RHΔ*ku80ΔompdcΔupΔrop21*::*HXGPRT*	RHΔ*ku80ΔompdcΔupΔhxgprt*	This paper
RHΔ*ku80ΔompdcΔupΔrop35*::*HXGPRT*	RHΔ*ku80ΔompdcΔupΔhxgprt*	This paper
RHΔ*ku80Δompdc*::*ROP35ΔupΔrop35*::*HXGPRT*	RHΔ*ku80ΔompdcΔupΔrop35*::*HXGPRT*	This paper
RHΔ*ku80ΔompdcΔupΔrop38*::*HXGPRT*	RHΔ*ku80ΔompdcΔupΔhxgprt*	This paper
PruΔ*ku80Δompdc*::*HXGPRT*	PruΔ*ku80Δhxgprt*	[14]
PruΔ*ku80Δrop5*::*HXGPRT*	PruΔ*ku80Δhxgprt*	[75]
PruΔ*ku80Δrop18*::*HXGPRT*	PruΔ*ku80Δhxgprt*	[75]

**Fig 6:**
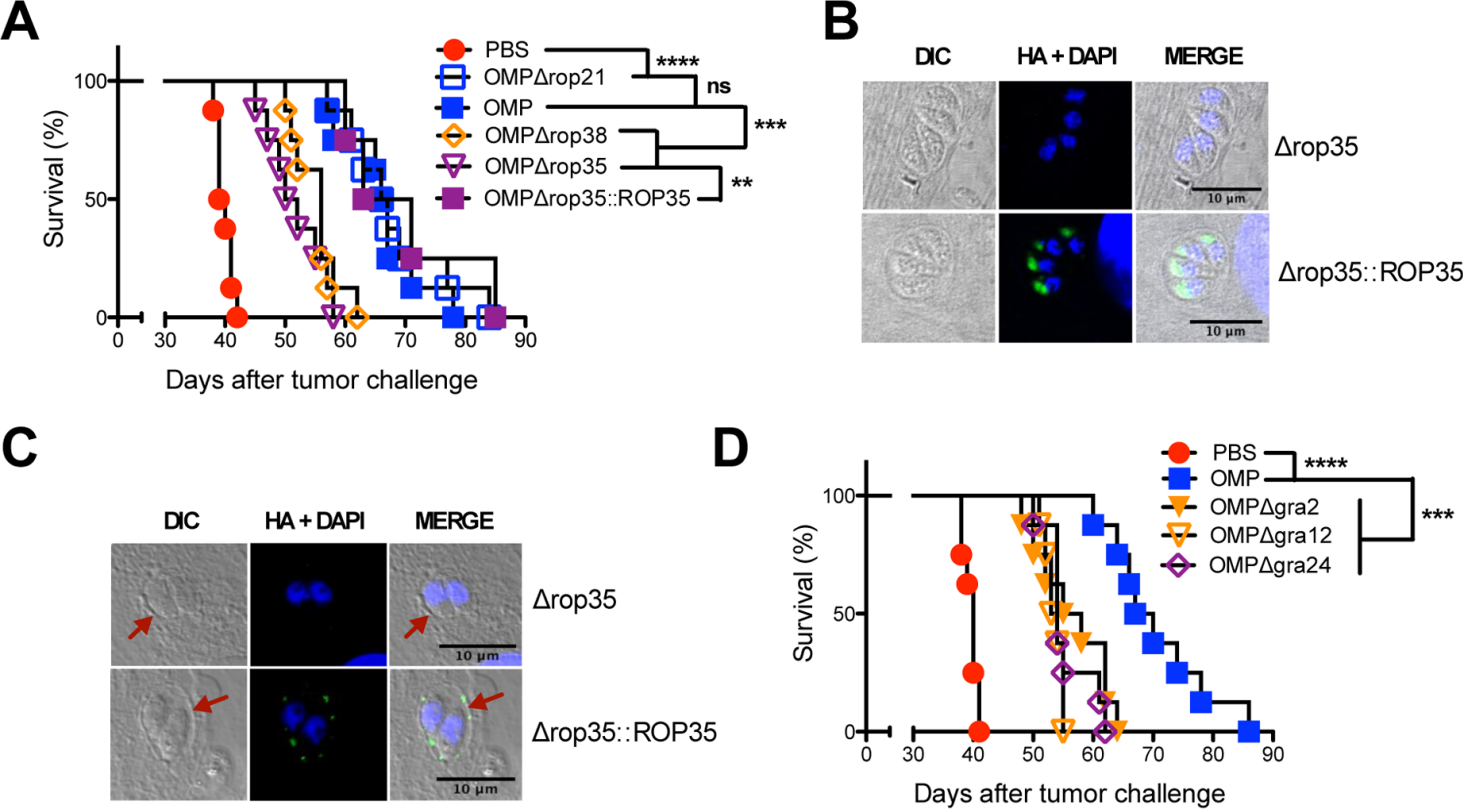
Secreted rhoptry proteins ROP35 and ROP38 and dense granule proteins GRA2, GRA12, and GRA24 are required for the antitumor response. (A) ID8DV ovarian tumors were established in C57BL/6 mice and groups of mice were treated with PBS or were vaccinated i.p. with tachyzoites of uracil auxotrophs using the three-dose treatment schedule. PBS treated or vaccinated i.p. with OMP or uracil auxotrophs lacking rhoptry proteins ROP21, ROP35, or ROP38, or vaccinated with a ROP35 complemented strain. (B) Validation of apical rhoptry localization of expressed C-terminal HA-tagged ROP35 in the complemented OMPΔ*rop35*::*ROP35* strain. DAPI stains the nuclei of both parasites and the host HFF cells they invaded. Localization of the HA tag (revealed by green fluorescence) is associated with the apical rhoptry organelles. Vacuole locations in the host cell are shown by differential interference contrast (DIC) microscopy. (C) PVM localization of ROP35. Parasites were allowed to invaded HFF cells for 14 h and after fixation the cytosolic surface of the PVM was exposed using 0.002% digitonin to expose the cytosolic surface of the PVM and ROP35 was localized. Vacuole locations (PVM) in the host cell are shown by differential interference contrast (DIC) microscopy (red arrowheads). The ROP35 HA tag is associated with the PVM. (D) ID8DV ovarian tumors were established in C57BL/6 mice and groups of mice were treated with PBS or were vaccinated i.p. with tachyzoites of uracil auxotrophs using the three-dose treatment schedule. PBS treated or vaccinated i.p. with OMP or uracil auxotrophs lacking dense granule proteins GRA2, GRA12, or GRA24. Data is representative of two independent experiments. ns was not significant, **p<0.01, ***P<0.001, ****P<0.0001.

We targeted the deletion of predicted active ROP kinases ROP21, ROP35 and ROP38. The deletion of ROP35 or ROP38 in the less virulent type II background was recently shown to abrogate latent infection without affecting parasite virulence during acute infection [75]. ROP35 and ROP38 are differentially expressed (>16 fold) between parasite strains and overexpression of ROP38 down regulates transcription of many host genes involved in proliferation, MAP kinase (MAPK) signaling, and apoptosis [78]. Deletion of ROP21 (OMPΔ*rop21*) did not affect the antitumor response (Fig 6A). In contrast, deletion of the ROP38 gene locus (OMPΔ*rop38*) as well as ROP35 (OMPΔ*rop35*) markedly impaired the antitumor response (Fig 6A).

The ROP35 deleted uracil auxotroph (OMPΔ*rop35*) was complemented with the wild-type ROP35 allele to generate strain OMPΔ*rop35*::*ROP35* by targeted insertion of ROP35 (C-terminal HA tagged) along with the cytosine deaminase gene into the *OMPDC* locus, and the complemented strain was selected by growth in cytosine which is converted to uracil by cytosine deaminase (CD) (S7 Fig) [105]. Importantly, the CD gene provides a new genetic marker for positive selection, which can be used specifically in uracil auxotrophic backgrounds without affecting parasite virulence. In addition, the CD marker can also be deployed as a genetic marker for negative selection by selection with the nontoxic prodrug 5-fluorocytosine [105]. Complementation of the ROP35 deletion by expression of a HA-tagged allele of ROP35 (OMPΔ*rop35*::*ROP35*) (Fig 6A) rescued the antitumor response (Fig 6A). Complementation of OMPΔ*rop38* was not currently feasible due to the existence of multiple related ROP38 alleles in the *ROP38* gene locus and the absence of any complete cosmid, fosmid, or BAC that spans this gene locus (ToxoDB.org) [75].

ROP21 and ROP38 were previously shown to localize to the PVM following their injection into host cells [78], however, the localization of ROP35 has not been reported. To determine whether ROP35 is also localized to the PVM after its injection into the host cell we used digitonin selective permeable conditions to expose the cytosolic face of the PVM [59,62] and found that ROP35 was localized to the PVM (Fig 6C). These data suggest that specific parasite secreted ROP effectors associated with the PVM and host transcriptional manipulation, but not specifically associated with parasite virulence, were involved in the mechanism that triggers the antitumor response.

Shortly after host cell invasion parasite dense granules massively secrete a large repertoire of proteins into the PV lumen and a subset of dense granule proteins including GRA2 [106] and GRA12 [107] establish association with an intravacuolar network (IVN) of nanotubular membranes. Deletion of IVN associated GRA proteins GRA2 (OMPΔ*gra2*) or GRA12 (OMPΔ*gra12*) markedly impaired the antitumor response (Fig 6D). In contrast, deletion of PVM associated GRA3 (OMPΔ*gra3*) did not affect the antitumor response (S8A Fig). Other GRA proteins such as GRA16 and GRA24 are secreted past the PVM to the host cell nucleus [80]. GRA24 deletion (OMPΔ*gra24*) markedly impaired the antitumor response (Fig 6D). In contrast, deletion of GRA16 (OMPΔ*gra16*), a secreted effector molecule that targets to the host nucleus and modulates host p53 and cell cycle [79], did not affect the antitumor response (S8A Fig). In addition, deletion of GRA15 (OMPΔ*gra15*) or ROP16 (OMPΔ*rop16*) did not affect the antitumor response (S8B Fig). Since GRA15 and ROP16 were previously shown to modulate various host transcriptional pathways [86,108,109], we also developed a double-targeted deletion of ROP16 and GRA15 (OMPΔ*gra15Δrop16*). The OMPΔ*gra15Δrop16* double mutant also exhibited a normal antitumor response (S8B Fig). Thus certain parasite secreted effector molecules that manipulate IL-12 (ROP16, GRA15) or that associate with the PVM (GRA3, ROP21) did not influence the antitumor response. In contrast, PVM associated ROP proteins ROP35 and ROP38, IVN-associated GRA proteins GRA2 and GRA12, as well as host nucleus-associated GRA24 were essential to the mechanism that triggers potent antitumor immunity.

### Parasite secreted PVM associated rhoptry molecules ROP5, ROP17, and ROP18 trigger the antitumor response

**Fig 7:**
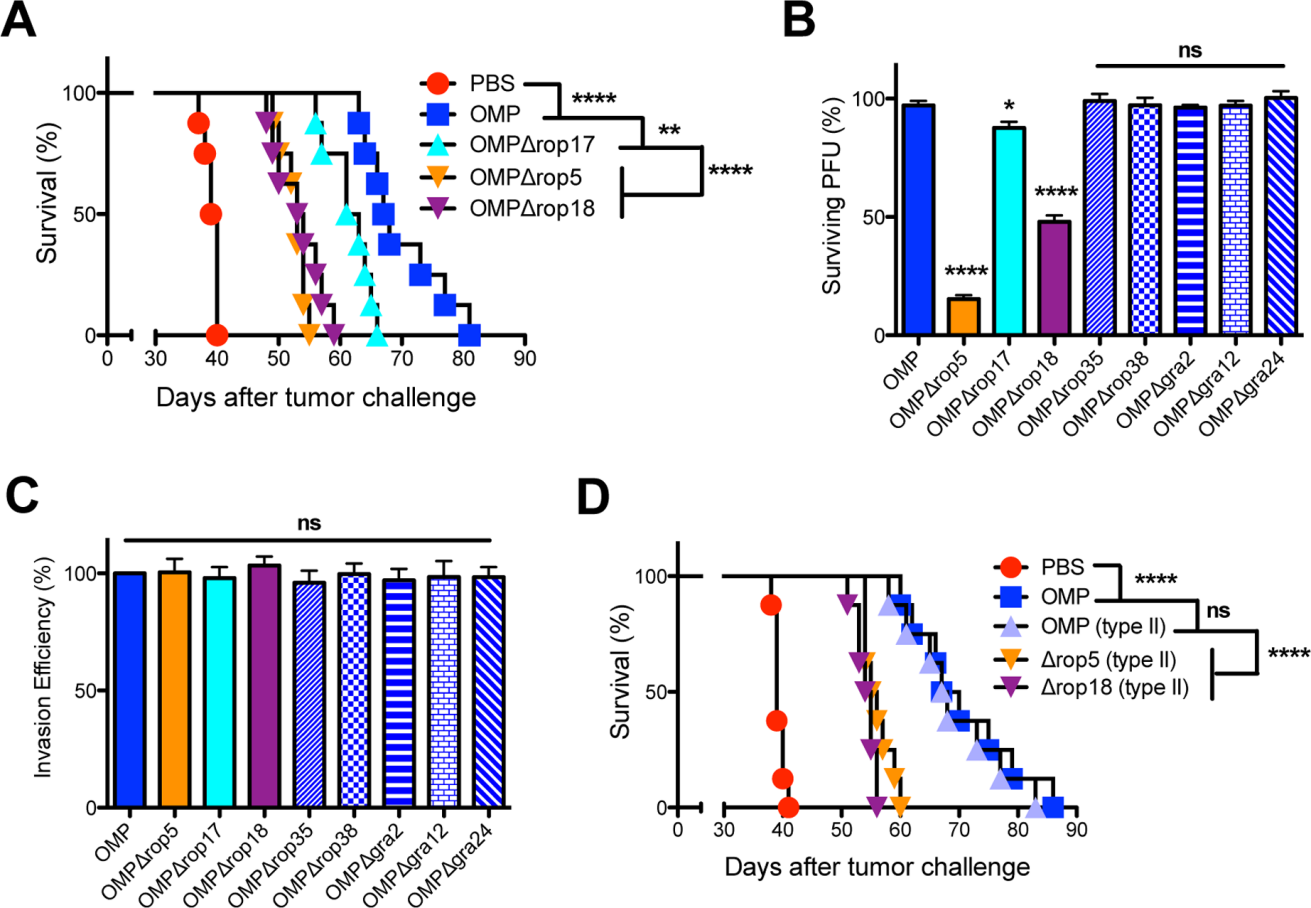
Rhoptry proteins ROP5, ROP17, and ROP18 are required for the antitumor response. (A) ID8DV ovarian tumors were established in C57BL/6 mice and groups of mice were treated with PBS, or were vaccinated i.p. with tachyzoites of uracil auxotrophs (OMP), or were vaccinated i.p. with uracil auxotrophs (OMP) lacking rhoptry proteins ROP5, ROP17, or ROP18 using the three-dose treatment schedule. (B) Mouse embryonic fibroblasts were stimulated with IFN-γ and parasite survival (measured as PFU) was determined for uracil auxotrophs mutants lacking specific rhoptry or dense granule proteins. (C) Relative parasite invasion efficiency of uracil auxotrophs was measured in MEFs. The parasite to PFU ratios (invasion efficiency) was measured in at least 4 independent assays and compared to the invasion efficiency of the parental OMP strain. (D) ID8DV ovarian tumors were established in C57BL/6 mice and groups of mice were treated with PBS, or were vaccinated i.p. with tachyzoites of type I uracil auxotrophs (OMP), or were vaccinated i.p. with type II OMP, or were vaccinated i.p. with type II strains lacking ROP5 or ROP18 using the three-dose treatment schedule. Data is representative of at least two independent experiments. ns was not significant, *p<0.05, **p<0.01, ****P<0.0001.

Since GRA2 was previously suggested to play a role in reducing association of IRGs to the PVM [66], we assessed whether other parasite molecules involved in resistance to IRGs were necessary to trigger the antitumor response. PVM associated protein complexes containing ROP5, ROP17, or ROP18 molecules resist host IRGs [62]. Deletion of ROP5 (OMPΔ*rop5*) or ROP18 (OMPΔ*rop18*) markedly impaired the antitumor response in comparison to parental OMP, or to ROP17 deletion (OMPΔ*rop17*), although ROP17 deletion itself also slightly impaired antitumor response (Fig 7A). This profile was consistent with a potential role for IRG resistance in mediating the antitumor response.

We assessed the resistance of the type I uracil auxotroph mutants to IRGs in IFN-γ stimulated mouse embryonic fibroblasts (MEFs). The OMPΔ*rop5* and OMPΔ*rop18* mutants, as expected, were markedly impaired in their survival in IFN-γ-activated MEFs (Fig 7B). Also as expected, the OMPΔ*rop17* mutant exhibited only a minor defect in IRG resistance and survival. However, other uracil auxotrophic mutants (OMPΔ*rop35*, OMPΔ*rop38*, OMPΔ*gra2*, OMPΔ*gra12*, OMPΔ*gra24*) that exhibited major defects in triggering the antitumor response fully resisted IRG mediated killing in IFN-γ activated MEFs (Fig 7B). Moreover, these uracil auxotrophic mutants did not exhibit any defects in their invasion of host cells (Fig 7C). In comparison, uracil auxotrophic mutants that exhibited no defects in their antitumor response retained their normal ability to resist IRG killing in IFN-γ stimulated MEFs (S9 Fig). Together, these results suggested that while ROP5, ROP17, and ROP18 functions were important for the antitumor response, resistance to IRGs did not appear to be specifically associated with the antitumor response.

Through genetic crosses, type I ROP5 has been previously associated with markedly increased virulence, whereas type II ROP5 was proposed to be avirulent [61,69]. Consistent with these findings, previous experiments have shown that the expression of virulent type I ROP5 in the less virulent type II strain increased parasite virulence by ~3 to 4-logs [66], and that type II *T*. *gondii* strains poorly resisted IRG killing mechanisms in IFN-γ activated cells [44]. We recently reported that the genetic deletion of ROP5 or ROP18 in the less virulent type II strain reduced parasite virulence by ~ 2-logs and correspondingly reduced resistance to killing by host IRGs in IFN-γ stimulated MEFs by ~10% [75]. To further assess the relationship of the antitumor response and IRG resistance we examined a type II uracil auxotroph (type II OMP) [14] as well as attenuated type II mutants lacking genes for ROP5 or ROP18 [75]. Type II OMP [14] (Table 1) elicited indistinguishable antitumor responses in comparison to type I OMP (Fig 7D). In addition, the deletion of ROP5 or ROP18 in the type II background markedly impaired the antitumor response (Fig 7D).

### PVM association is essential but the ROP18 kinase activity is dispensable for triggering the antitumor response

**Fig 8:**
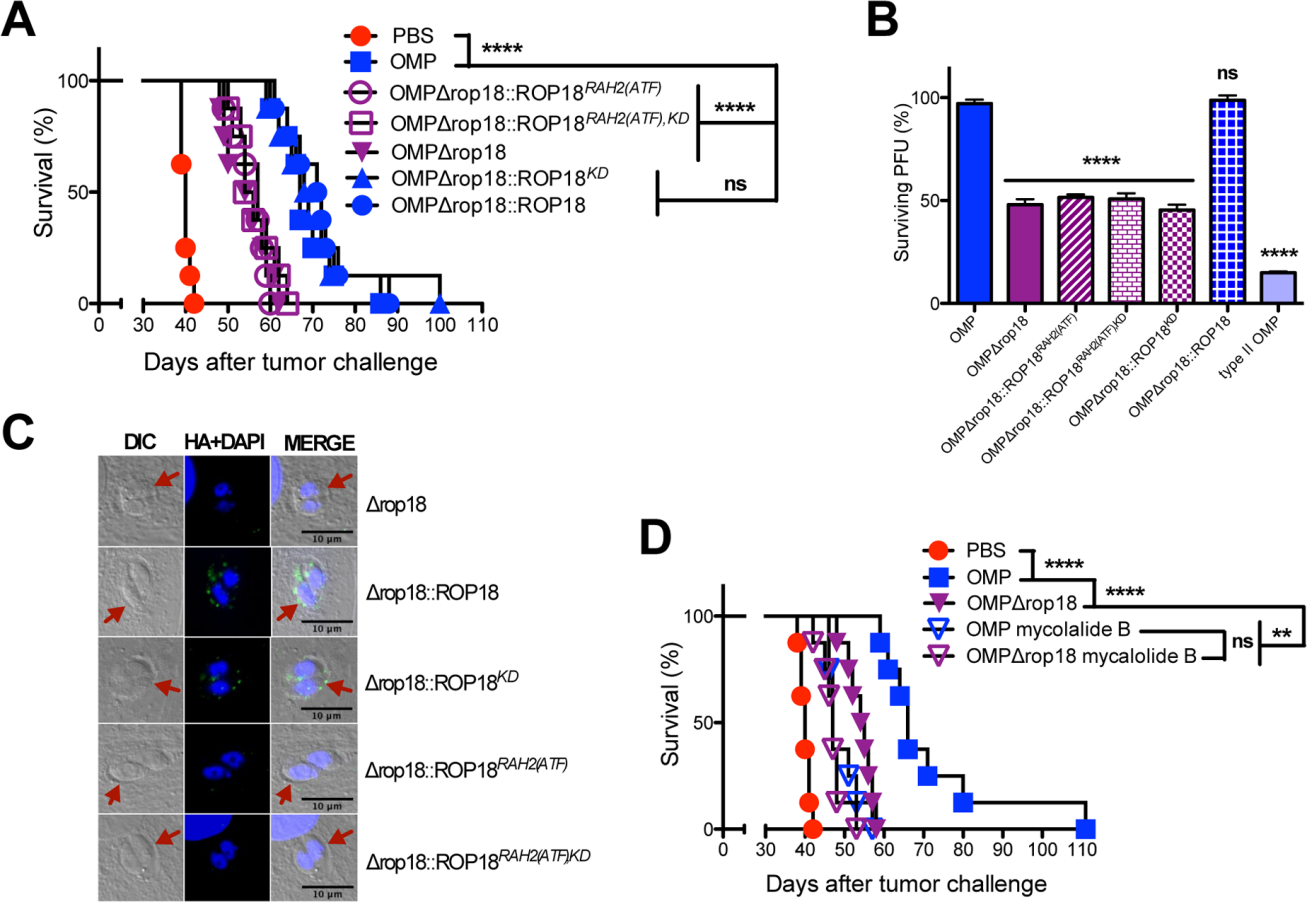
ROP18 PVM association is required for the antitumor response but the kinase function of ROP18 is dispensable. (A) ID8DV ovarian tumors were established in C57BL/6 mice and groups of mice were treated with PBS, or were vaccinated i.p. with tachyzoites of uracil auxotrophs (OMP), OMPΔ*rop18*, or were vaccinated i.p. with OMPΔ*rop18* uracil auxotrophs that were complemented with wild-type or mutant ROP18 gene alleles using the three-dose treatment schedule. (B) Mouse embryonic fibroblasts were stimulated with IFN-γ and parasite survival (measured as PFU) was determined for type I and type II uracil auxotrophs and type I uracil auxotrophs expressing wild-type or mutant alleles of ROP18. (C) PVM localization of wild-type and mutant ROP18 proteins. Parasites were allowed to invaded HFF cells for 14 h and after fixation the cytosolic surface of the PVM was exposed using 0.002% digitonin and HA tagged ROP18 was localized. Vacuole locations (PVM) in the host cell are shown by differential interference contrast (DIC) microscopy (red arrowheads). Wild-type and the kinase-dead ROP18 mutant localized to the PVM, however, deletion of the *RAH2(ATF)* domain abolished PVM association. (D) ID8DV ovarian tumors were established in C57BL/6 mice and groups of mice were treated with PBS, or were vaccinated i.p. with tachyzoites of uracil auxotrophs (OMP) or OMPΔ*rop18*, or were vaccinated i.p. with mycalolide B treated tachyzoites of uracil auxotrophs (OMP) or OMPΔ*rop18* using the three-dose treatment schedule. Data is representative of at least two independent experiments. ns was not significant, **P<0.01, ****P<0.0001.

To further examine the role of resistance to IRG killing mechanisms from other potential mechanisms we focused on the active ROP18 kinase that directly phosphorylates IRG effectors to inactivate host innate immunity [63]. We complemented the ROP18 deleted OMPΔ*rop18* strain by expression of wild-type (OMPΔ*rop18*::ROP18) or various mutant C-terminally HA-tagged ROP18 alleles, (OMPΔ*rop18*::*ROP18*^*KD*^, OMPΔ*rop18*::*ROP18*^*RAH2(ATF)*^, or the double mutant OMPΔ*rop18*::*ROP18*^*RAH2(ATF)*,*KD*^) (S10A Fig) via targeted insertion of the ROP18 gene along with the cytosine deaminase gene into the *OMPDC* locus through positive selection in cytosine (S7 Fig). Complementation of OMPΔ*rop18* with C-terminal HA-tagged wild-type ROP18 (OMPΔ*rop18*::*ROP18*) rescued the antitumor response (Fig 8A). Remarkably, complementation of OMPΔ*rop18* with C-terminal HA-tagged kinase-dead (*KD*) ROP18 (OMPΔ*rop18*::*ROP18*^*KD*^) [63] also rescued the antitumor response (Fig 8A), suggesting that the ROP18 virulence function mediated by the ROP18 kinase activity was not essential for the antitumor response.

The ROP18 ATF6β association domain is identical to the second arginine rich amphipathic helix (RAH2) domain of ROP18 that targets ROP18 to the PVM after its injection into host cells [64,110]. While active wild-type ROP18 as well as kinase-dead ROP18 fully rescued the antitumor response, complementation of OMPΔ*rop18* with C-terminal HA-tagged *RAH2(ATF)* deficient alleles (designated as *RAH2(ATF)* [75]) of ROP18 (OMPΔ*rop18*::*ROP18*^*RAH2(ATF)*^ or the double mutant OMPΔ*rop18*::*ROP18*^*RAH2(ATF)*,*KD*^ failed to rescue the antitumor response (Fig 8A).

Previous studies have shown that ectopic expression of ROP18 kinase activity in the absence of infection and PVM formation mediated the degradation of host NF-κβ [76] as well as host ATF6β [77]. In addition to ROP18 kinase activity, ROP18 PVM association is also required for resistance to host IRGs and virulence [63,64]. Therefore, we measured the resistance of mutant ROP18 uracil auxotrophs to IRGs and PV killing. The wild-type allele of ROP18 fully rescued resistance to IRG killing in the ROP18 deficient strain, however, the kinase-dead as well as *RAH2(ATF)* domain deleted ROP18 alleles, as expected, failed to rescue any detectable resistance to IRG killing (Fig 8B). These results were corroborated in *ex vivo* experiments using IFN-γ stimulated bone marrow derived macrophages. The *RAH2(ATF)* domain deleted and the kinase-dead ROP18 alleles failed to confer resistance to host IRGs in IFN-γ stimulated macrophages, whereas the wild-type ROP18 allele was resistant to PV killing (S10B Fig). In addition, the type II OMP uracil auxotroph that elicits a potent antitumor response indistinguishable from type I OMP (Fig 7D), as expected, was exquisitely susceptible to IRG mediated killing in comparison to type I OMP (Fig 8B).

To further assess the requirement for the association of ROP18 with the PVM to trigger the antitumor response, we localized wild-type ROP18 as well as the kinase-dead ROP18 to the cytosolic surface of newly formed PVs using digitonin selective permeable conditions (Fig 8C). In contrast, deletion of the *RAH2(ATF)* domain, as expected, completely abolished the association of ROP18 with the PVM after its injection into host cells (Fig 8C). These data suggested that the antitumor response was not dependent on the ROP18 kinase activity but did require ROP18 PVM association.

We next asked whether injection of ROP18 into bystander host cells in the absence of invasion could trigger the antitumor response. To address this question we compared the antitumor efficacy of vaccinations using the ROP18 deleted OMP strain (OMPΔ*rop18*) compared to mycalolide B treatment of this strain. A significant increase in resistance to tumor development was observed in ROP18 deficient OMP parasites compared to mycalolide B treated ROP18 deficient OMP parasites (Fig 8D). However, no significant difference in the antitumor response was observed between mycalolide B treated OMP compared to mycalolide B treated ROP18 deficient OMP parasites (Fig 8D). These results suggest that ROP18 kinase activity in the absence of PVM association did not trigger the antitumor response.

## Discussion

Our studies extend the application of reverse genetic engineering in *Toxoplasma gondii* by using a genetically tractable Δ*ku80* uracil auxotrophic vaccine background to reliably generate parasite strains with precisely targeted gene deletions of secreted parasite effectors. Targeted deletions of specific rhoptry and dense granule proteins in isogenic uracil auxotrophic backgrounds enabled a comparable measurement of the functional contribution of each of these secreted effectors in triggering the antitumor response to ID8DV ovarian tumors. These genetic studies revealed key roles for ROP5, ROP17, ROP18, ROP35, ROP38, GRA2, GRA12, and GRA24 secreted parasite effectors in triggering the antitumor response.

Host cell invasion by *T*. *gondii* requires motility and secretion from apical organelles [111]. Chemical treatment of *T*. *gondii* with 4-bromophenacyl bromide (4BPB) irreversibly inhibits rhoptry secretion and invasion [97–99]. The antitumor response was absent in 4BPB treated uracil auxotrophs. Mycalolide B treated parasites fail to invade host cells due to a motility defect but can still secrete from their rhoptry organelles [100,101]. Our mycalolide B inhibition experiments suggested that rhoptry secretion in the absence of host cell invasion provided a detectable, though suboptimal, antitumor response. However, this antitumor response was markedly increased through a sequential combination of rhoptry secretion, host cell invasion, and the formation of the PV and PVM. Collectively, these findings demonstrate that secreted GRA proteins and PVM associated ROP proteins trigger a potent antitumor response from within invaded host cells in the tumor microenvironment.

Therapeutic vaccination of solid tumors with uracil auxotrophs converts tolerogenic tumor microenvironments into immune stimulatory microenvironments in aggressive models of murine pancreatic cancer, melanoma, and ovarian cancer through the stimulation of tumor-specific CD8^+^ T cell populations [15,16,19]. Induction of the antitumor response in primary tumors did not require CD4^+^ T cell populations in B16 melanoma [15] or disseminated pancreatic tumors [19], although in treated mice that survived the primary cancer, CD4^+^ T cells were shown to play an important role in enforcing immunity to pancreatic cancer [20]. Our data suggests that in addition to CD8^+^ T cells [16,17], MHCII and CD4^+^ T cells were also necessary to mediate the antitumor response induced by uracil auxotrophs to ID8DV ovarian tumors.

Uracil auxotrophic mutants were previously shown to preferentially target and invade CD11c^+^ antigen presenting cells in the ID8DV tumor microenvironment [16]. Our findings show that heat killed uracil auxotrophs failed to elicit an antitumor response. In addition, noninvasive heat killed uracil auxotrophs induced early IL-12 but, surprisingly, did not stimulate IFN-γ in the tumor microenvironment, suggesting that T cell populations were not highly activated in the absence of parasite invasion. Parasite extracts also failed to elicit any detectable antitumor response, reinforcing the importance of parasite invasion as a key step in the antitumor mechanism. Live invasive parasites rapidly induced high levels of IL-12 followed shortly thereafter by high-level production of IFN-γ. Moreover, high IFN-γ levels present after therapeutic vaccination with live parasites were associated with the effective clearance of intracellular uracil auxotrophs in the parasite invaded CD11c^+^ cell population within 66 h. Collectively, these findings indicate that parasite invasion of CD11c^+^ antigen presenting cells rapidly triggered the antitumor response through IFN-γ producing CD4^+^ and CD8^+^ T cell populations.

High-level expression of IL-12 is triggered in CD8α^+^ dendritic cells by MyD88 dependent signaling downstream of TLR11/12 dependent recognition of parasite profilin [37,38,89]. However, uracil auxotroph vaccination of MyD88 knockout (*MyD88*^*-/-*^) mice was previously shown to elicit a reduced IL-12 response that was sufficient to drive IFN-γ production by protective T cell populations [10]. Our findings revealed that the antitumor response was intact in *MyD88*^*-/-*^ mice, suggesting that the primary MyD88 dependent pathway for high level IL-12 production by CD8α^+^ dendritic cells was not strictly required. However, mice completely deficient in CD8α^+^ dendritic cells (*Batf3*^*-/-*^ or *IRF8*^*-/-*^ knockout mice) do not produce protective IL-12 or IFN-γ and rapidly succumb to *T*. *gondii* infection [36,112], and our experiments (present work) revealed that CD8α^+^ dendritic cell deficiency in *Batf3*^*-/-*^ knockout mice abrogated the antitumor response. These findings establish the essentiality of the CD11c^+^ CD8α^+^ dendritic cell in the antitumor response elicited by live uracil auxotrophs.

CD11c^+^ CD8α^+^ dendritic cells have been recognized play an important role in cross presentation of antigens to prime CD8^+^ T cell mediated immunity to tumors [113]. We have previously shown that the invasion of ID8DV tumor associated immune suppressed CD11c^+^ antigen presenting cells by uracil auxotrophs triggered the expression of the T-cell receptor costimulatory molecules CD80 and CD86 [16,17]. Invasion of CD11c^+^ antigen presenting cells by uracil auxotrophs also increased CD80 and CD86 expression in melanoma and pancreatic cancer tumor microenvironments [15,19], and uracil auxotroph invasion of dendritic cells in naive mice increased the expression of CD80 and CD86 as well as MHCI that is required to initiate antigen presentation that drives the development of protective T cell populations [4]. The high level induction of CD86 by *T*. *gondii* was previously shown to require active parasite invasion and host cell signaling through the JNK pathway [114]. Consistent with increased antigen presentation by uracil auxotroph invaded CD11c^+^ cells, antigen presenting cells harvested 18 h after uracil auxotroph vaccination of the ID8DV ovarian tumor microenvironment were previously shown to have regained a substantial ability to present the ovalbumin (OVA OT-I) peptide on MHCI for recognition by OVA-specific CD8^+^ T cells [16]. These findings showed that uracil auxotrophs reversed immune suppression in CD11c^+^ antigen presenting cells in the immune suppressed ID8DV tumor microenvironment [16–18]. The constitutive activation of the host unfolded protein response ER stress pathway mediated by XBP-1 in ID8DV ovarian tumor associated dendritic cells was recently shown to be a central immunosuppressive mechanism that promotes ovarian cancer progression by dampening antigen presentation [115]. Moreover, targeted deletion or inactivation of XBP-1 to quench the unfolded protein response to stress was demonstrated to restore the immunostimulatory activity of ID8DV tumor associated CD11c^+^ dendritic cells and promoted the development of antitumor CD8^+^ T cell populations [115]. *T*. *gondii* has been shown to manipulate the unfolded protein response through ATF6β [77]. These findings suggest that additional studies are necessary to further address whether the antitumor mechanism elicited by uracil auxotrophs targets host cell stress pathways.

We used recently engineered Δ*ku80* genetic models [92,103] to target parasite gene deletions to identify secreted parasite molecules that were required for the antitumor response. PVM associated ROP5/ROP17/ROP18 parasite effector molecules play a central role in mediating the antitumor response. In virulent type I parasite strains, ROP5 associates in high molecular weight complexes with either ROP17 or ROP18 to mediate IRG resistance and virulence mechanisms [61–63,69]. The kinase activity of ROP17 [62] as well as ROP18 [63] is required for IRG resistance and parasite virulence. In addition to kinase activity, PVM association of the ROP5/ROP17/ROP18 complexes has been shown to be essential for IRG resistance and parasite virulence [62–64]. Our findings show that while PVM association of ROP18 was essential to trigger the antitumor response, the ROP18 kinase activity was not required for this response. Since deletion of ROP5 also abrogated the antitumor response, it seems likely that the antitumor mechanism is mediated through the ROP5/ROP18 PVM complex. In addition to IRG resistance, the kinase activity of ectopically expressed ROP18 in the absence of invasion and the PVM has been previously shown to mediate the degradation of the host NF-κβ p65 subunit [76] as well as the host ER stress sensor ATF6β [77]. It remains to be determined whether a PVM associated kinase inactive ROP18 could associate with host ATF6β. In addition to previously recognized associations of ROP18 with host IRGs, host NF-κβ [76], and ATF6β [77], ROP18 was previously shown in yeast two-hybrid assays to associate with additional host cell proteins [116,117]. Further studies would be necessary to decipher whether the interaction of ROP18 with any of these host proteins triggers the antitumor mechanism.

Our studies show that PVM associated ROP35 and ROP38 molecules were also essential to the antitumor response. The function of ROP35 is poorly understood although this molecule, like ROP38, exhibits strain type specific variation in mRNA expression levels [78]. ROP38 has been previously shown to modulate the expression level of a large number of host genes involved in MAPK signaling cascades, apoptosis, and host cell proliferation [78]. In addition, the ROP38 gene locus [78] as well as ROP35, were recently shown to mediate the parasites ability to establish latent infection through a mechanism that did not affect parasite virulence [75]. In addition, ROP35 and ROP38 molecules were not identified in ROP5/ROP17/ROP18 associated protein complexes [62]. These data suggest that the ROP35 and ROP38 antitumor mechanisms most likely function independently of the ROP5/ROP17/ROP18 complexes. Additional studies are thus essential to decipher the ROP35 and ROP38 antitumor mechanisms.

We identified GRA24 (also known as TgBRADIN [118]) as a secreted dense granule molecule that was crucial to trigger the antitumor response. GRA24 was previously shown to traffic past the PVM to the host cell nucleus and this effector molecule functions to sustain the activation of host cell p38α MAPK by both type I and type II parasite strains [87]. In addition, host cell modulation of IL-12 by GRA24 was previously shown to be parasite strain type dependent and while GRA24 in type II strains stimulated IL-12 production, GRA24 in type I strains did not [87]. Our results show that type I and type II uracil auxotrophs elicited equally potent antitumor responses. These findings suggest that the GRA24 antitumor mechanism is potentially associated with p38α MAPK signaling.

The secreted GRA15 molecule was previously shown to elicit IL-12 in type II strain infection but not during infection by type I strains [86,100,109]. Type II GRA15 increased host cell IL-12 production through strain type dependent manipulation of host NF-κβ signaling [86,100]. In contrast, type I ROP16, but not type II ROP16, has been shown to suppress IL-12 production [83,109]. Our experiments revealed that neither GRA15 or ROP16 were essential for the antitumor response. GRA15 and ROP16 effectors have been previously identified as key molecules that underpin the induction of the M2 phenotype in macrophages polarized by infection with type I parasites, whereas these secreted molecules were associated with the induction of the classical activation M1-like phenotype in macrophages polarized by infection with type II parasites [109,119]. In contrast, we previously reported that type I uracil auxotrophs induced a unique profile of macrophage polarization exhibiting features of both the M1 as well as the M2 polarized phenotypes [120]. In addition, vaccination with nonreplicating type I uracil auxotrophs quickly elicited IL-12 in naive mice [7] as well as after therapeutic vaccination of established tumor microenvironments (present work) [15,16,19]. In contrast, infection with virulent type I strains was previously shown to suppress the early production of IL-12 [121]. Together these findings suggest that parasite strain type dependent host cell modulation by certain parasite secreted effectors after vaccination by nonreplicating uracil auxotrophs is mechanistically distinct from host modulation that occurs following infection by replicating parasite strains. Further studies are therefore necessary to understand how, in the absence of parasite replication, secreted effectors that are known to be associated with virulence function to trigger antitumor immunity.

In addition to secreted rhoptry effectors and the host nucleus targeted GRA24, we identified an important role for IVN associated dense granule proteins GRA2 and GRA12 in the antitumor response. Deletion of GRA2 was previously shown to increase IRG coating of the PVM [66], to slightly reduce parasite virulence [122], to modulate the presentation of PV membrane bound or soluble PV lumen antigens by MHCI in parasite invaded macrophages and dendritic cells [123], and to also induce a morphological loss of the appearance of the IVN within the PV luminal spaces without affecting the association of host ER and mitochondria with the PVM [106]. The morphology and dynamic membranes of the IVN are poorly characterized, however, recent helium ion microscopy studies have suggested that IVN structures can extend from the surface of the tachyzoite stage parasite in the PV directly to IVN junctions on the PV lumen side of the PVM [124]. Our findings suggest that the role of GRA2 in formation of the IVN [106,123] was essential for the antitumor response. In addition, GRA2 deletion was recently shown to reduce parasite heterophagy, a parasite process that ingests host cell cytoplasm into the lumen of the PV [125]. While the heterophagy mechanism has not yet been precisely determined, it is highly likely that this process involves the PVM, which serves as a membrane barrier between the PV lumen and the host cell cytoplasm. One previously proposed model of the PVM has suggested that invaginations of the PVM could develop into PVM tubules that extend into the PV luminal space [110]. This model hypothesized that these tubular network structures may preferentially attract molecules from the cytosolic face of the PVM that have an affinity for high negative curvature, and then retain these PVM molecules through potential binding partners associated with IVN tubular membranes [110]. While the function of GRA12 is currently unknown, like GRA2, this molecule also localizes to the IVN structures in the PV space [107]. Remarkably, GRA12 was previously shown to associate specifically with the ROP5/ROP18 complexes that assemble on the PVM [62]. Collectively, these findings suggest that the antitumor mechanism depends on maintenance of dynamic interactions between the IVN and the PVM. Further studies of the parasite IVN and PVM would be necessary to identify the specific mechanisms that underpin the antitumor response mediated by the IVN associated GRA2 and GRA12 dense granule secreted molecules. Our results argue that secretion of rhoptry effector proteins, host cell invasion, formation of the PV, secretion of specific GRA effector proteins to the IVN structures present in the PV lumen, and the export of GRA protein(s) past the PVM to the host cell nucleus coordinately triggers the potent ID8DV antitumor immune response elicited by nonreplicating uracil auxotroph mutants of *T*. *gondii*.

## Materials and Methods

### Ethics statement

All procedures involving mice were in accordance with the guidelines published by the Guide for the Care and Use of Laboratory Animals of the National Institute of Health. All animal experiments were reviewed and approved by the Institutional Animal Care and Use Committee of Dartmouth College under protocols bzik.dj.1 and bzik.dj.2. All procedures involving mice were reviewed and approved by the Institutional Animal Care and Use Committee (IACUC) of Dartmouth College (Animal Welfare Assurance Number #3259–01) and were in accordance with the guidelines published in the Guide for the Care and Use of Laboratory Animals of the National Institutes of Health.

### Mice

Female 7–9 week old C57BL/6, *IL-12p40*^*-/-*^, *IL-12p35*^*-/-*^, *MyD88*^*-/-*^, *Batf3*^*-/-*^, *IFN-γ*^*-/-*^, *CD8*^*-/-*^, and *MHCII*^*-/-*^ mice were purchased from Jackson laboratories (Bar Harbor, ME) and were maintained under specific pathogen-free conditions at the Center for Comparative Medicine and Research at the Geisel School of Medicine at Dartmouth.

### Cells

ID8 cells were provided by Katherine Robey (University of Kansas Medical Center) and were transduced with *Defβ29* and *Vegf-A* to establish the ID8-*Defβ29*/*Vegf-A* (ID8DV) xenograft ovarian tumor model in the C57BL/6 background [90]. ID8DV cells were cultured in Dulbecco's Modified Eagle's Medium (DMEM)/high glucose (HyClone) in 10% fetal bovine serum (FBS) (Invitrogen) and Penicillin and Streptomycin (Corning) (final concentration 100 units/ml Penicillin and 100 μg/ml Streptomycin. Human foreskin fibroblasts (HFFs) were cultured in Eagle's Minimal Essential Medium (EMEM) (Lonza) in 10% FBS and Antimycotic-Antibiotic (Gibco) (final concentration 100 units/ml Penicillin,100 μg/ml Streptomycin, and 25 μg/ml Fungizone). Mouse embryonic fibroblasts (MEFs) in the C57BL/6 background were obtained from the ATCC and were cultured in DMEM in 15% FBS and Penicillin and Streptomycin.

### Parasites

*Toxoplasma gondii* was maintained by serial passage of tachyzoites in HFF monolayers cultured in EMEM containing 1% FBS, (2 mM glutamine, 100 units/ml penicillin, and 100 μg/ml streptomycin) as previously described [7]. Uracil auxotrophs were supplemented with 0.2 mM uracil (Sigma-Aldrich). OMP [13], CPS [5], CPS-YFP [96], and other strains used for treatment of ID8DV tumors were cultured in HFF monolayers and freshly lysed extracellular tachyzoites were purified by filtration through 3.0 μm filters (Nuclepore), and washed extensively in Dulbecco's Phosphate Buffered Saline (PBS) prior to intraperitoneal injection in 0.2 ml PBS in ID8DV ovarian tumor-bearing mice.

### Xenograft model of ovarian carcinoma

Aggressive disseminated ovarian tumors were established in mice by intraperitoneal injection of 2 X 10^6^ ID8DV tumor cells [90]. Independent samples of identical ID8DV stocks were used for all experiments. Intraperitoneal treatment of established ID8DV tumors with uracil auxotrophs was performed using 2 x 10^6^ tachyzoites in a one-dose (25 d), two-dose (8, 20 d), three-dose (8, 20, 32 d), or five-dose (8, 20, 32, 44, 56 d) schedule after ID8DV tumor challenge. Each preparation of tachyzoites was subjected to a plaque forming unit (PFU) assay to measure parasite viability and verify treatment dose. Tumor-bearing mice were monitored for wellness and tumor development.

### Parasite survival assays

Parasite survival was measured in MEFs that were pre-stimulated for 24 h with murine IFN-γ (Peprotech) (final concentration of 100 units/ml). Triplicate MEF monolayers that were stimulated or not stimulated with IFN-γ in 24 well culture plates were infected with 100–200 PFU of each parasite strain and incubated for 6 days at 37°C. All PFU in each culture was scored using light microscopy and survival percentage was calculated as the number of PFU in IFN-γ stimulated MEFs divided by the number of PFU scored in nonstimulated MEFs (no IFN-γ treatment). Survival assays of parasite strains were performed in at least three independent experiments for each strain examined. C57BL/6 bone marrow derived macrophages were differentiated as previously described, and 4 x 10^6^ macrophages were seeded in 6-well trays overnight. Macrophages were pre-stimulated, or not stimulated, for 6 h with IFN-γ (100 U/ml, Preprotech) and TNF alpha (TNF-α) (10 U/ml, Preprotech) then cultures were infected with ~ 100 tachyzoites per well and cultures were incubated for 6 days to develop PFU. Cultures were stained with coomassie brilliant blue to identify PFU. Survival percentage was calculated as the number of PFU in IFN-γ stimulated macrophages divided by the number of PFU scored in nonstimulated macrophages. Survival assays of parasite strains were performed in at least two independent experiments for each strain examined.

### Replication and invasion assays

The intracellular growth rate of parasite strains was measured in HFF cells in a 30 h growth assay using previously described methods [5]. Briefly, HFF monolayers were infection at a multiplicity of infection (MOI) of ~0.1. After 1 h of invasion the noninvaded parasites were removed by extensive (4X) washing in PBS and the number of parasites per vacuole was scored 30 h later from at least 50 vacuoles per sample. The relative rate of invasion of mutant strains was measured in MEFs in comparison to parental OMP. Two hundred tachyzoites of tested strains were added in triplicate to each monolayer of MEF cells in 24 well trays and allowed to invaded for 3 h. Noninvaded parasites were removed by extensive washing in PBS and the number of PFU per well was scored 6–7 days later by microscopy. Experiments were repeated in at least four independent assays.

### Heat killed parasites and extracts

Uracil auxotroph tachyzoites were filtered through 3 μm nuclepore membranes and washed extensively in PBS. Tachyzoites were heat killed at 56°C for 15 minutes as previously described [126]. Excreted-secreted antigen (ESA) extracts were prepared by incubation of 2 x 10^8^ tachyzoites for 3 h at 37°C in EMEM supplemented with 10% FBS followed by removal of tachyzoites by centrifugation at 1200*g* for 7 minutes as previously described [127]. Total *Toxoplasma* lysate antigen (TLA) extracts were prepared by sonication of tachyzoites on ice. Complete loss of viability was verified in PFU assays. Soluble tachyzoite antigen (STAg) extracts were prepared by high-speed centrifugation of TLA as previously described [39]. Parasite extracts were stored at -80°C. For each treatment, ID8DV tumor-bearing mice received the equivalent of 5 x 10^6^ tachyzoites of ESA, TLA, or STAg extracts.

### Chemical inhibition

Tachyzoites were purified using 3.0 μm Nuclepore filters, concentrated by centrifugation at 1000g and washed extensively in PBS. Tachyzoites (2 x 10^7^/ml) were treated with 100 μM 4-bromophenylacyl bromide (4BPB) (Sigma-Aldrich) for 10 min at 37°C and quenched with 20 volumes of complete media and washed in PBS [126]. Tachyzoites (2 x 10^7^/ml) in PBS were treated with 3 μM mycalolide B (Wako) for 15 min at 37°C and quenched with 20 volumes of complete media and washed in PBS [100,101]. Parasite inactivation of invasion was verified in PFU assays.

### Antibody depletions

Purified monoclonal antibodies αCD8 (2.43), αCD4 (GK1.5), and isotype control Rat IgG2a were purchased from BioXcell. For cell depletions, 500 μg of antibody was administered by intraperitoneal injection 1 day prior to and 250 μg of antibody was administered by intraperitoneal injection 0 and 3 d after each treatment with uracil auxotrophs. NK cells were depleted using αNK1.1 (PK136) antibody (a generous gift from Dr. Charles Sentman at Dartmouth, EBioscience), and intraperitoneal injections of 50 μg of αNK1.1 antibody were given on day -1, 0, and +3 relative to each treatment with uracil auxotrophs [19]. In all experiments target cell populations were depleted by greater than 98%.

### Cytokine assays

Peritoneal fluid obtained by peritoneal lavage using 5 ml PBS [16] was used for the detection of cytokines in the ovarian tumor microenvironment. IL-12p40 and IL-12p70 were determined using duplex luminex assays (Millipore). IFN-γ, IL-1α and IL-1β were measured using a mouse 32-plex Luminex assay (Millipore). Type I interferons were measured using a mouse IFN alpha and IFN beta 2Plex assay (EBioscience).

### Primers and PCR

*T*. *gondii* genomic DNA was purified from tachyzoites using the DNA Blood Mini Kit (Qiagen) on a robotic Qiacube (Qiagen). PCR products for targeting vector construction were amplified from primers (Integrated DNA Technologies) using High Fidelity polymerases (Roche). The *Toxoplasma* genome resource (www.toxodb.org) [128] was used to identify gene loci of interest and sequences to design oligonucleotide primers.

### Gene knockouts

Targeted ROP and GRA knockouts were generated in the OMP parental uracil auxotroph strain RHΔ*ku80ΔompdcΔupΔhxgprt* which was generated by the deletion of *HXGPRT* selectable marker from the uridine phosphorylase of OMP (strain RHΔ*ku80ΔompdcΔup*::*HXGPRT*) locus using 6-thioxanthine (250 μg/ml) (Acros Biochemicals) and uracil selection as previously described [13]. ROP and GRA gene targeting plasmids were developed in the pRS416 yeast shuttle vector using yeast recombination to fuse, in order, 3 distinct PCR products with 31 to 34 bp crossovers on DNA fragments that included a 5’ target gene flank, the *HXGPRT* selectable marker, and a 3’ target flank (S6 Fig). Gene targeting plasmids were engineered to delete the entire ROP or GRA gene locus including 400 bp of the immediate 5’ UTR. The DNA primers used to generate the 5' and 3' gene targeting flanks and the nucleotides deleted in each ROP and GRA knockout strain are shown in supplemental material (S1 Table). Targeting plasmids were linearized via a unique restriction site at the junction of the 5' targeting flank and pRS416 vector prior to transfection into RHΔ*ku80ΔompdcΔupΔhxgprt* and continuous selection in mycophenolic acid (25 μg/ml) and xanthine (250 μM) and uracil as previously described [13,14,92,103,104]. Isolates were subcloned 20 days after transfection by limiting dilution. Targeted knockouts were validated by genotype analysis using PCR assays (S6 Fig) to measure: (i) PCR 1, targeted deletion of the coding region of the targeted gene (DF and DR primers); (ii) PCR 2, correct targeted 5' integration (CXF & 5'DHFRCXR primers); and (iii) PCR 3, correct targeted 3' integration (3'DHFRCXF and CXR primers) using DNA validation primers shown in supplemental material (S2 Table).

### Complementation

Selected knockout strains were complemented with wild-type or mutant alleles of the deleted ROP gene. A new positive genetic selection was developed for uracil auxotroph backgrounds based on cytosine auxotrophy using the cytosine deaminase (*CD*) selectable marker that converts cytosine to uracil [105]. Genes for complementation analysis were C-terminally tagged with the HA peptide to allow fluorescent visualization of protein expression and the complementing genes were targeted for integration at the *OMPDC* gene locus that was already deleted for *OMPDC* and *HXGPRT* [13]. Complementation targeting plasmids were developed in the pRS416 yeast shuttle vector using yeast recombination to fuse, in order, a 5' *OMPDC* target flank, the complementing gene of interest plus the genes 5' UTR (~ 1 Kbp) synthesized on one or two PCR products, the CD selectable marker, and the 3' *OMPDC* target flank (S7 Fig). The DNA primers used to generate the complementing gene (synthesized on one two or three PCR products), the CD marker, and target flanks are shown in supplemental material (S1 Table and S3 Table). Mutant ROP18 alleles expressing a kinase-dead (KD) ROP18, or mutant ROP18 lacking the PVM and ATF6β association domain (RAH2ATF) were designed using previously described mutations [63,77]. Targeting plasmids were linearized via a unique restriction site at the junction of the 5' targeting flank and pRS416 vector prior to transfection. Following transfection parasites were grown 1 d in uracil medium, then were switched to selection medium containing 1 mM cytosine, and isolates were subcloned 30 days later by limiting dilution. Targeted knockouts were validated by genotype analysis using PCR assays (S7 Fig) to measure: (i) PCR 4, correct targeted 5' integration and (ii) PCR 5, correct targeted 3' integration of the complementing transgene and *CD* at the OMPDC locus using DNA validation primers (S4 Table).

### Immunofluorescence assays

HFF cells were cultured on circular micro cover glass (Electron Microscopy Sciences) and were infected with parasites for 18–30 h. For localization of rhoptry proteins to the rhoptry organelles, infected cells were fixed with Histochoice (Amresco), and permeabilized in 0.1% saponin (Sigma) for 10 min. For localization of rhoptry proteins to the PVM, infected cells were fixed in 4% paraformaldehyde and exposed to 0.002% digitonin for 5 minutes as previously described [62] to expose proteins associated with the cytosolic face of the PVM. All samples were blocked with 10% FBS and incubated with a 1:500 dilution of primary rabbit monoclonal α-HA-tag antibodies (Cell Signaling) 1 h at room temperature. Preparations were washed 3 times with PBS and incubated 1 h at RT with a 1:1000 dilution of secondary goat anti-rabbit IgG antibodies conjugated to Alexa Fluor 488. Samples were mounted in Slowfade Gold antifade with DAPI (Life Technologies) and then imaged with a Nikon A1R SI confocal microscope (Nikon, Inc.). Confocal images were processed with FIJI [129]. Vacuole locations were determined by differential interference contrast (DIC) microscopy.

### Flow cytometry

Antibodies used were CD16/CD32 (93, ebioscience), APC-Cy7-conjugated anti-mouse CD45 (30-F11, Biolegend), and AF647-conjugated anti-mouse CD11c (N418, Biolegend). FACS analysis was performed using a Miltenyi 8-color MACSQuant and data was analyzed using FlowJo v9.77 and v10 (TreeStar).

### Statistical analysis

Statistical analysis was performed using PRISM software (Graphpad Software). Statistical significance in acute virulence assays in mice was determined using the Log-rank Mantel-Cox test performed using the Gehan-Breslow-Wilcoxon test. *P* values less than or equal to 0.05 (*P*<0.05) were considered to be significant. All other statistic analysis was performed using an unpaired two-tailed Students t test with the assumption of equal variance, and a *P* value less than or equal to 0.05 (*P*<0.05) was considered to be significant.

## Supporting Information

S1 Fig Intravenous treatment of ID8DV tumors. (A) ID8DV ovarian tumors were established in C57BL/6 mice and groups of mice were treated with PBS, or were vaccinated i.p. with tachyzoites of OMP uracil auxotrophs, or were vaccinated i.v. with tachyzoites of OMP uracil auxotrophs using the three-dose treatment schedule. (B) Groups of mice were vaccinated with 2 x 10^6^ tachyzoites of OMP two weeks apart (VAC), or were treated with PBS (naive). Twelve months later vaccinated or age-matched naive mice were challenged with 5,000 tachyzoites of the virulent RH strain and survival was monitored. Data is representative of two independent experiments. ****P<0.0001.
(TIF)

S2 Fig Antitumor responses measured in *IL-12p40*^*-/-*^ knockout mice. ID8DV ovarian tumors were established and groups of mice were treated with PBS or were vaccinated i.p. with tachyzoites of OMP uracil auxotrophs using the three-dose treatment schedule. (A) Wild-type or *IL-12p40*^*-/-*^ C57BL/6 mice. ns was not significant, **P<0.01.
(TIF)

S3 Fig NK cells and MHCII play divergent roles in the antitumor response. ID8DV ovarian tumors were established in wild-type or *MHC-II*^*-/-*^ mice and groups of mice were treated with PBS or vaccinated i.p. with tachyzoites of OMP uracil auxotrophs using the standard three-dose treatment schedule. (A) Mice were depleted of NK cells using αNK1.1 antibody. (B) Results from *MHCII*^*-/-*^ mice. Data is representative of two independent experiments. ns was not significant, **P<0.01.
(TIF)

S4 Fig Heat killed parasites do not invade or significantly stimulate type I interferons. (A) Heat killed parasites were assayed for invasion and PFU formation compared to no heat inactivation. (B) IFN-β production in the tumor microenvironment following treatment with intact or heat killed uracil auxotrophs. (C) IL-1α and IL-1β production in the tumor microenvironment following treatment with heat killed or invasive uracil auxotrophs. Experiments were performed using the CPS-YFP expressing uracil auxotroph. Data is representative of two independent experiments. ns was not significant, **P<0.01, ****P<0.0001.
(TIF)

S5 Fig Mycalolide B and 4-bromophenylacyl bromide (4BPB) treatment inactivates infectivity. Tachyzoites of OMP uracil auxotrophs were treated with 4BPB, mycalolide B, or were left untreated and infectivity was determined in PFU assays. Parasites were allowed to attach and invade for 12 h prior to rinsing and initiation of PFU assays. Data is representative of three independent experiments. ****P<0.0001.
(TIF)

S6 Fig Development of uracil auxotrophic strains lacking specific GRA or ROP proteins. Mutants were selected in mycophenolic acid (MPA) + xanthine (X) + uracil selection medium and were validated for knockout genotype using PCR1, PCR2, and PCR3 as shown.
(TIF)

S7 Fig Uracil auxotrophic mutants were complemented by selection with cytosine deaminase. The HA-tagged complementing gene and its ~1 Kbp 5' UTR region was placed next to the bacterial cytosine deaminase (*CD*) gene (flanked by the 5' and 3' dhfr upstream regions [105]), and both genes were flanked by 5' and 3' *OMPDC* locus targeting flanks for insertion of the complementing gene and the *CD* marker at the *OMPDC* locus. Complemented strains were selected in medium containing mycophenolic acid (MPA) + xanthine (X) + cytosine and genotypes were verified using PCR 4 and PCR 5 as shown.
(TIF)

S8 Fig ID8DV ovarian tumors were established in C57BL/6 mice and groups of mice were treated with PBS, or were vaccinated i.p. with tachyzoites of uracil auxotrophs, or (A) were vaccinated i.p. with OMP or OMP lacking GRA proteins GRA3 or GRA16, or (B) vaccinated i.p. with OMP or OMP lacking GRA15 or ROP16, or GRA15 and ROP16. Data is representative of two independent experiments. ****P<0.0001.
(TIF)

S9 Fig Uracil auxotrophic mutants that do not affect the antitumor response survive in IFN-γ activated mouse embryonic fibroblasts. PFU survival was measured in IFN-γ activated MEFs infected with uracil auxotrophic vaccine mutants that did not affect the antitumor response. ns was not significant
(TIF)

S10 Fig (A) Rhoptry localization of expressed ROP18 gene alleles. Immunofluorescence validation of apical rhoptry localization of expressed C-terminal HA-tagged ROP18 alleles in complemented OMPΔ*rop18* strains: wild-type ROP18 (OMPΔ*rop18*::*ROP18*), a kinase-dead (*KD*) ROP18 (OMPΔ*rop18*::*ROP18*^*KD*^), a RAH2 domain deleted ROP18 (OMPΔ*rop18*::*ROP18*^*RAH2(ATF)*^), or a kinase-dead and *RAH2(ATF)* mutant (OMPΔ*rop18*::*ROP18* ^*RAH2(ATF)*,*KD*^*)*. DAPI stains the nuclei of both parasites and the host cells they invaded. The HA tag (revealed) by green fluorescence associated with apical rhoptry organelles is present in the complemented ROP18 strains and is absence in the ROP18 deleted strain. Vacuole locations are shown by differential interference contrast (DIC) microscopy. (B) Bone marrow derived macrophages were stimulated with IFN-γ and TNF-α and parasite survival (measured as PFU) was determined for complemented OMPΔ*rop18* strains: wild-type ROP18 (OMPΔ*rop18*::*ROP18*), a kinase-dead (*KD*) ROP18 (OMPΔ*rop18*::*ROP18*^*KD*^), or a RAH2 domain deleted ROP18 (OMPΔ*rop18*::*ROP18*^*RAH2(ATF)*^).
(TIF)

S1 Table Oligonucleotide primers used for development of GRA or ROP knockout plasmids. Primer pairs used to create 5' and 3' gene targeting flanks. The chromosomal location of each gene and nucleotides targeted for deletion are shown for each plasmid construct.
(DOCX)

S2 Table Oligonucleotide primers used to validate ΔGRA and ΔROP knockouts. (DOCX)

S3 Table Oligonucleotide primers used for construction of complementation targeting plasmids. Complementing genes were C-terminally HA tagged and were constructed using one or two PCR segments as shown.
(DOCX)

S4 Table Oligonucleotide primers used to validate targeted insertion of complementing genes at the OMPDC locus. (DOCX)
