# Supplementary figures and images for: Secretion of Rhoptry and Dense Granule Effector Proteins by Nonreplicating Toxoplasma gondii Uracil Auxotrophs Controls the Development of Antitumor Immunity
Source: PLoS Genet. 2016 Jul 22;12(7):e1006189. doi: 10.1371/journal.pgen.1006189 (PMC4957766; doi:10.1371/journal.pgen.1006189)

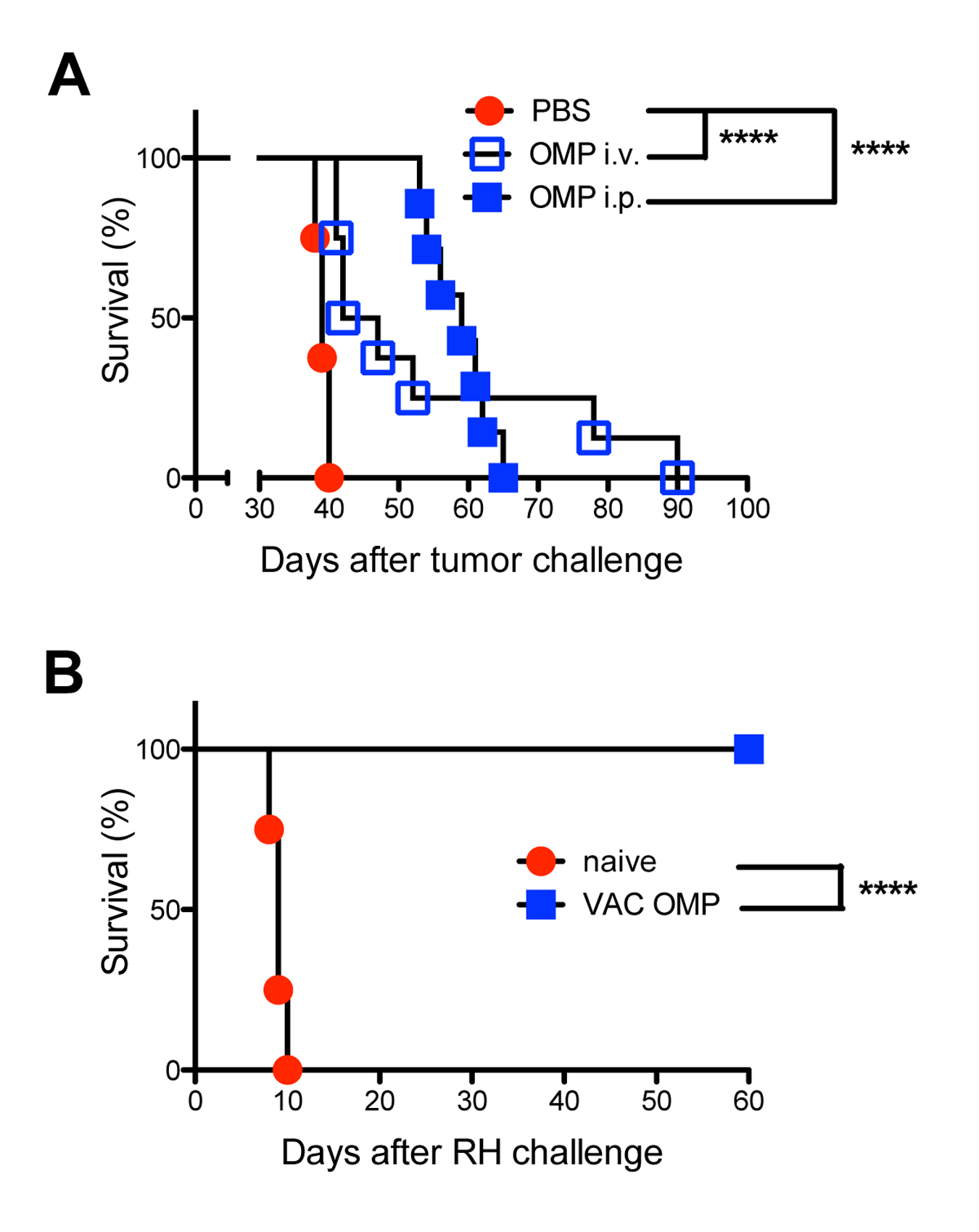

Supplement: S1 Fig — (A) ID8DV ovarian tumors were established in C57BL/6 mice and groups of mice were treated with PBS, or were vaccinated i.p. with tachyzoites of OMP uracil auxotrophs, or were vaccinated i.v. with tachyzoites of OMP uracil auxotrophs using the three-dose treatment schedule. (B) Groups of mice were vaccinated with 2 x 10^6^ tachyzoites of OMP two weeks apart (VAC), or were treated with PBS (naive). Twelve months later vaccinated or age-matched naive mice were challenged with 5,000 tachyzoites of the virulent RH strain and survival was monitored. Data is representative of two independent experiments. ****P<0.0001. (TIF) [file pgen.1006189.s001.tif]

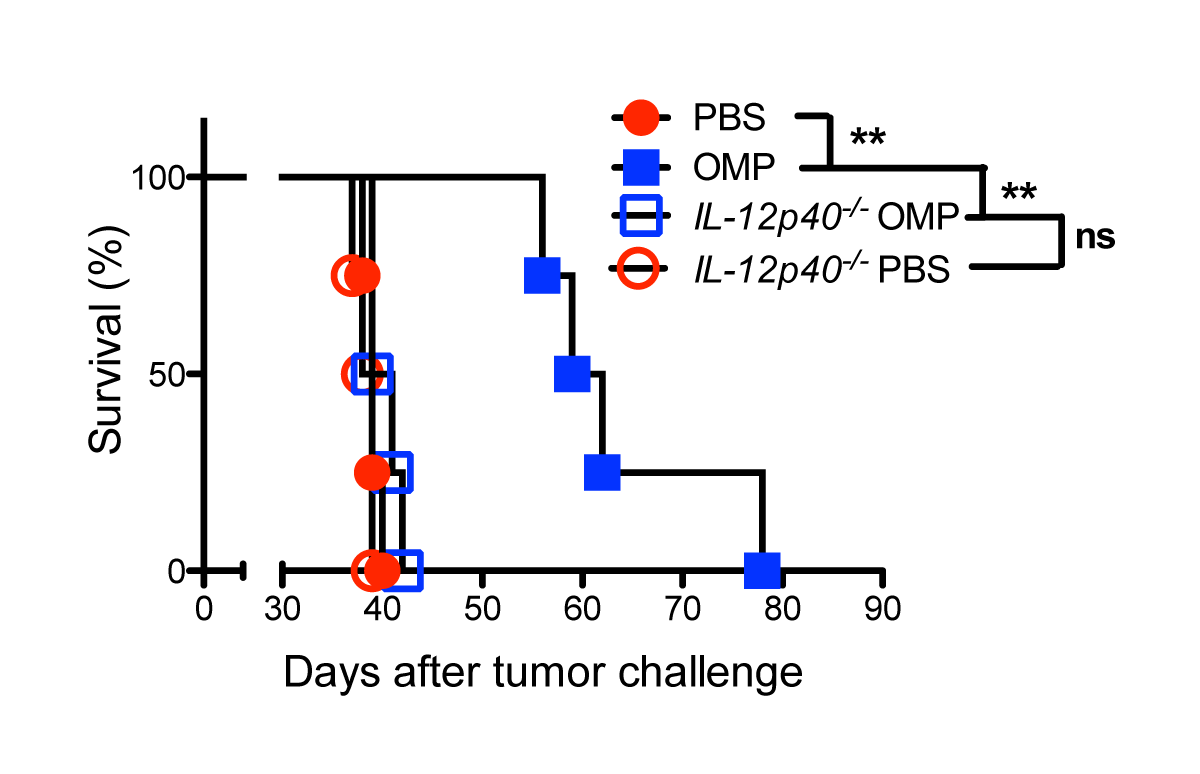

Supplement: S2 Fig — ID8DV ovarian tumors were established and groups of mice were treated with PBS or were vaccinated i.p. with tachyzoites of OMP uracil auxotrophs using the three-dose treatment schedule. (A) Wild-type or IL-12p40-/- C57BL/6 mice. ns was not significant, **P<0.01. (TIF) [file pgen.1006189.s002.tif]

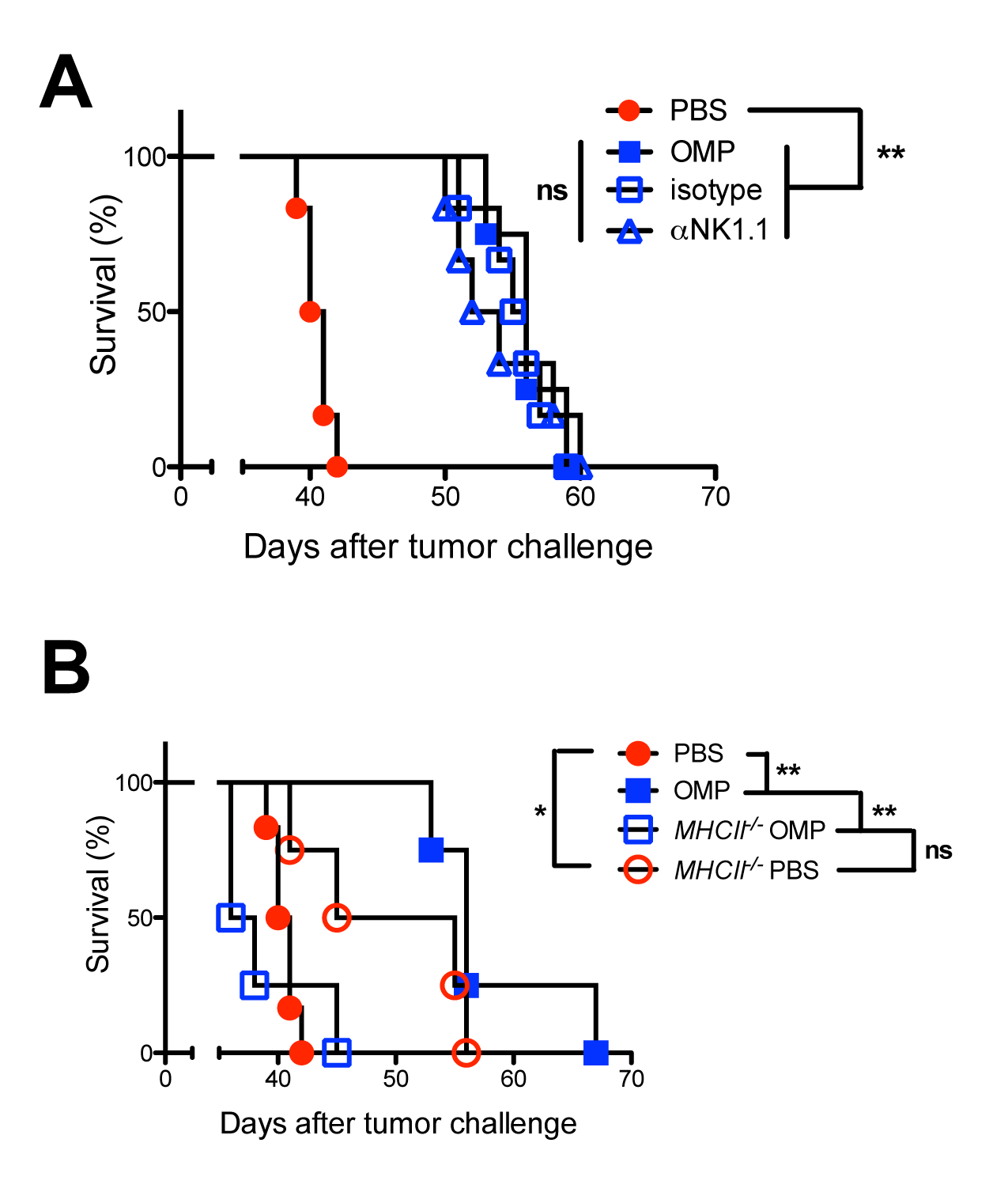

Supplement: S3 Fig — ID8DV ovarian tumors were established in wild-type or MHC-II-/- mice and groups of mice were treated with PBS or vaccinated i.p. with tachyzoites of OMP uracil auxotrophs using the standard three-dose treatment schedule. (A) Mice were depleted of NK cells using αNK1.1 antibody. (B) Results from MHCII-/- mice. Data is representative of two independent experiments. ns was not significant, **P<0.01. (TIF) [file pgen.1006189.s003.tif]

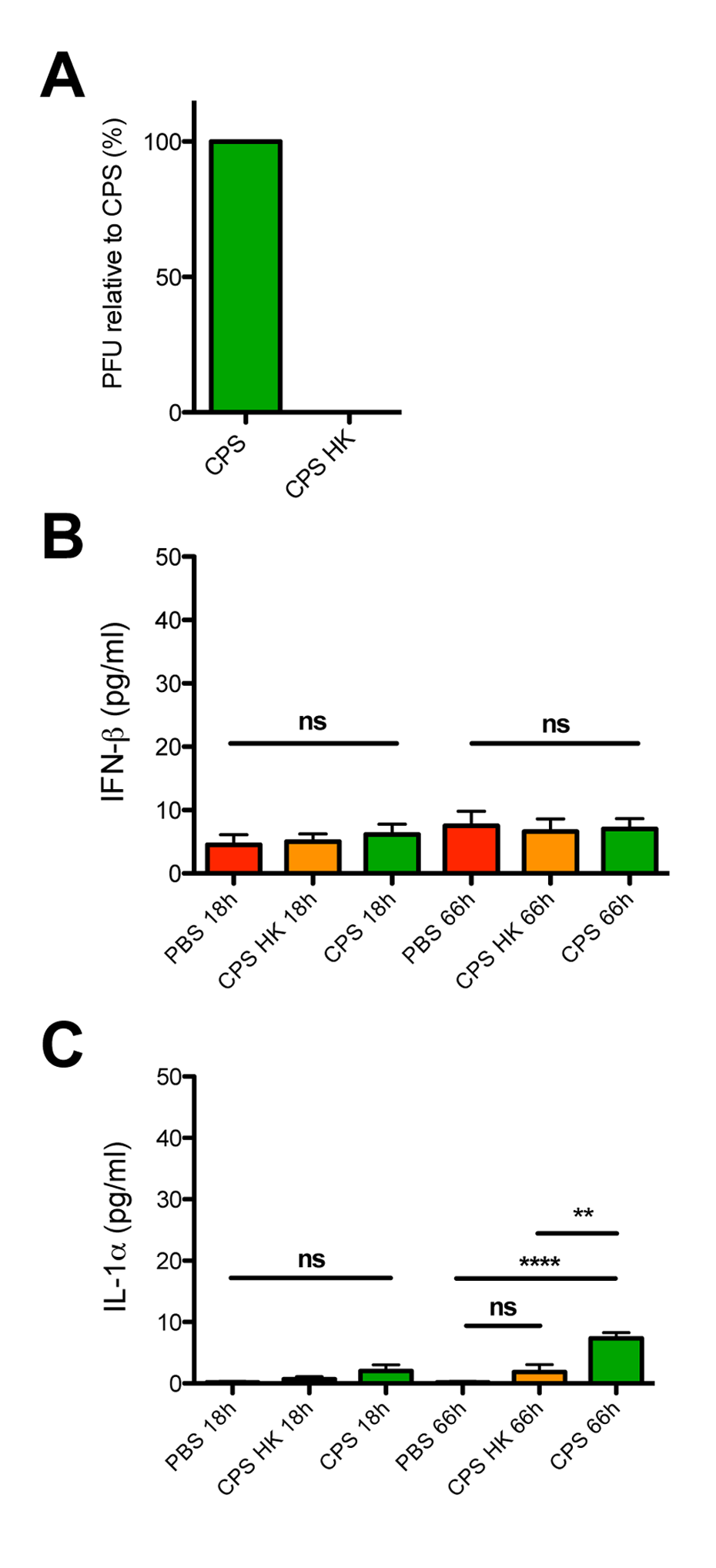

Supplement: S4 Fig — (A) Heat killed parasites were assayed for invasion and PFU formation compared to no heat inactivation. (B) IFN-β production in the tumor microenvironment following treatment with intact or heat killed uracil auxotrophs. (C) IL-1α and IL-1β production in the tumor microenvironment following treatment with heat killed or invasive uracil auxotrophs. Experiments were performed using the CPS-YFP expressing uracil auxotroph. Data is representative of two independent experiments. ns was not significant, **P<0.01, ****P<0.0001. (TIF) [file pgen.1006189.s004.tif]

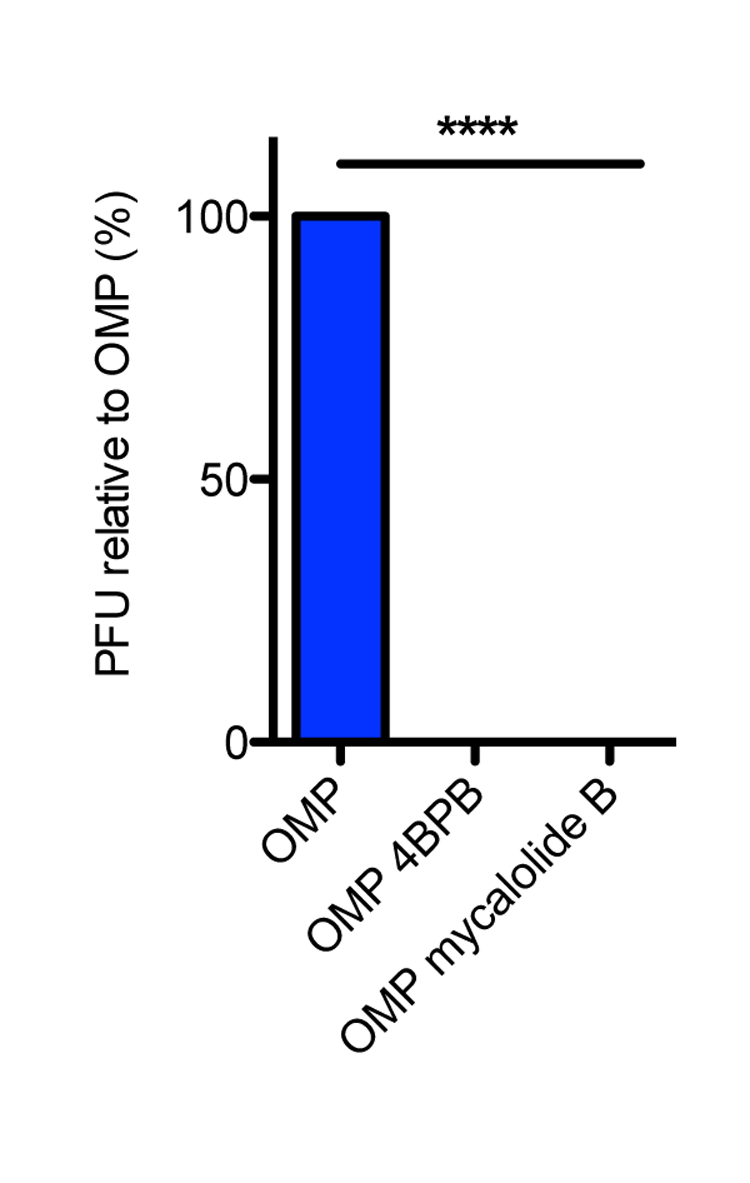

Supplement: S5 Fig — Tachyzoites of OMP uracil auxotrophs were treated with 4BPB, mycalolide B, or were left untreated and infectivity was determined in PFU assays. Parasites were allowed to attach and invade for 12 h prior to rinsing and initiation of PFU assays. Data is representative of three independent experiments. ****P<0.0001. (TIF) [file pgen.1006189.s005.tif]

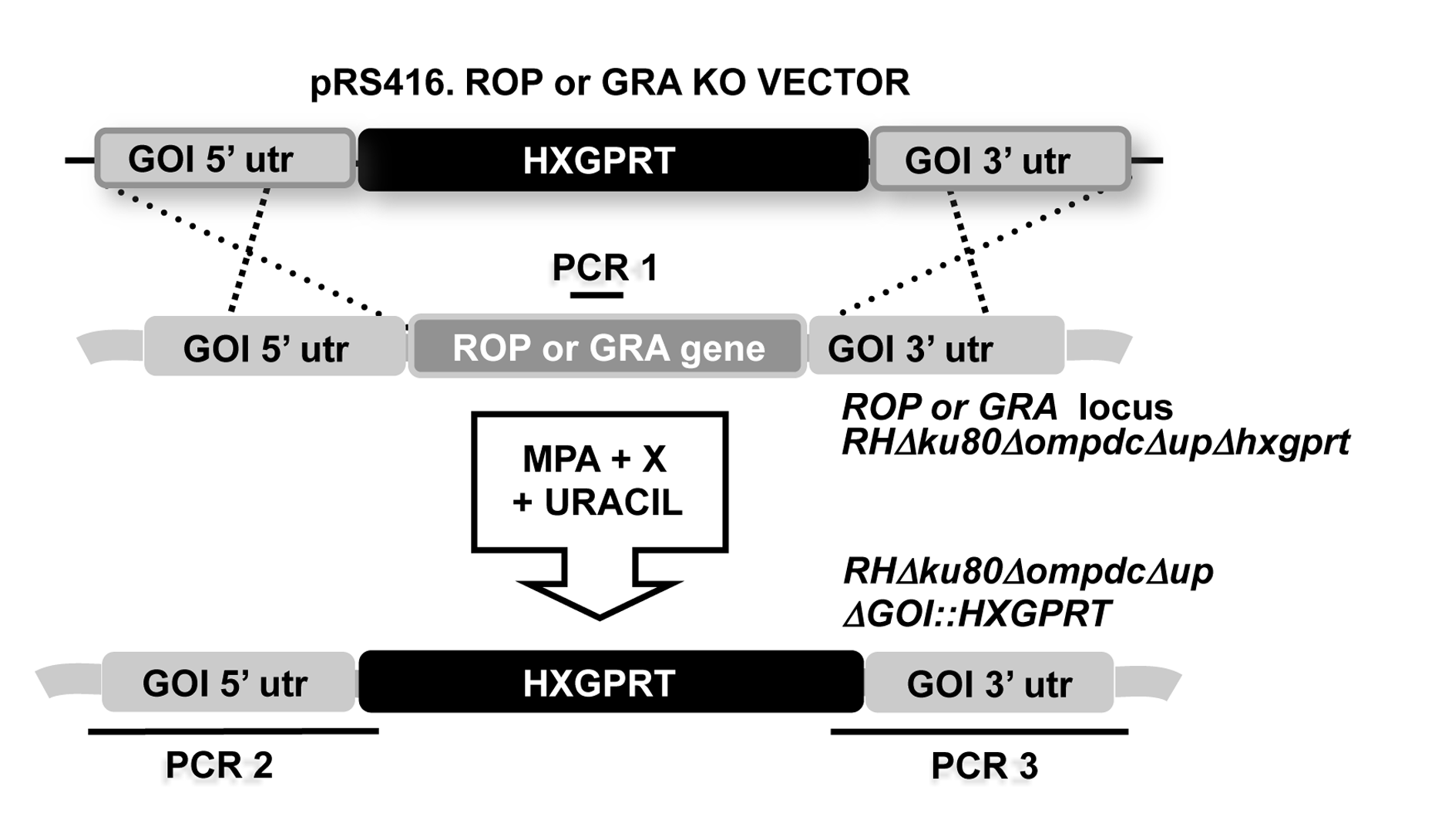

Supplement: S6 Fig — Mutants were selected in mycophenolic acid (MPA) + xanthine (X) + uracil selection medium and were validated for knockout genotype using PCR1, PCR2, and PCR3 as shown. (TIF) [file pgen.1006189.s006.tif]

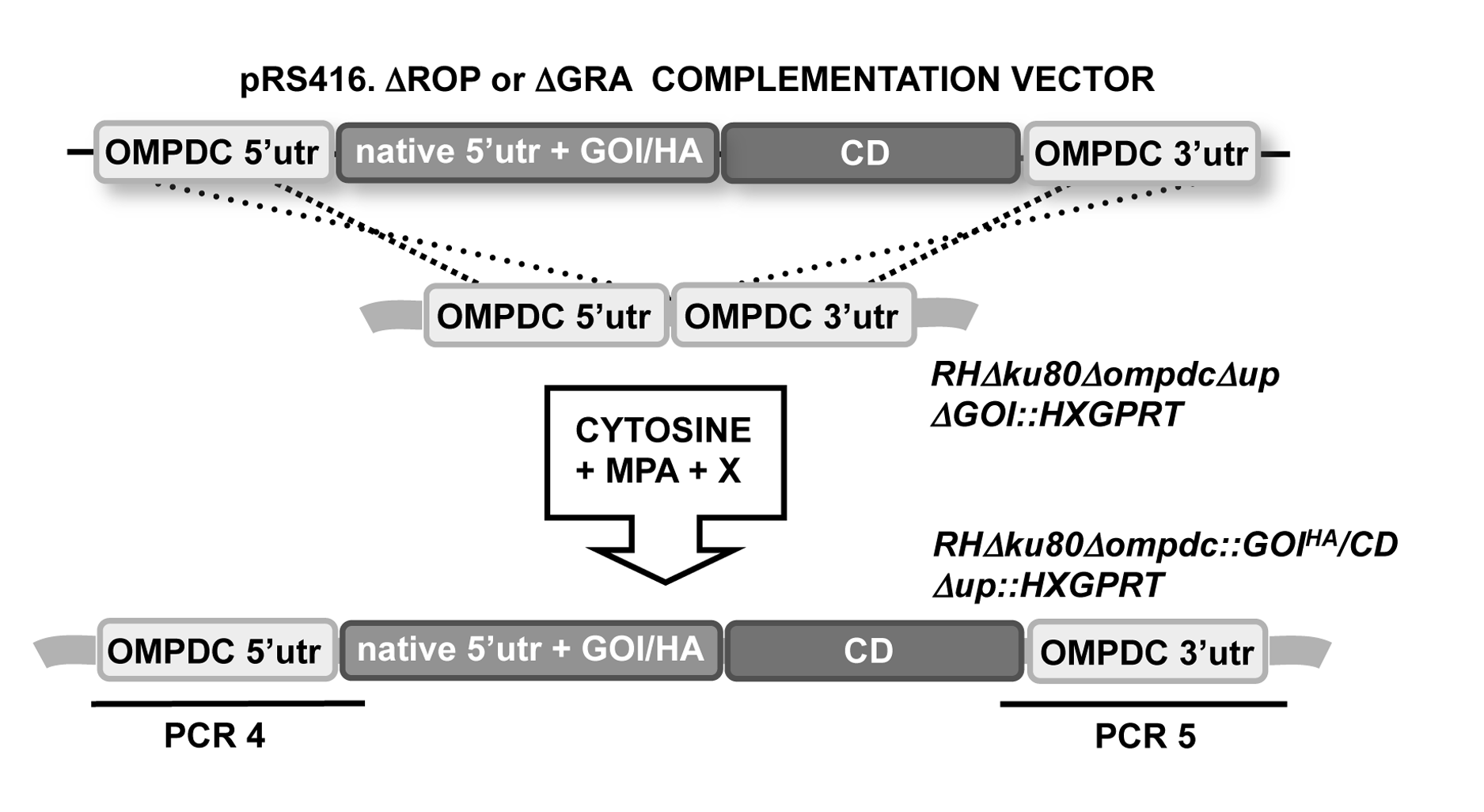

Supplement: S7 Fig — The HA-tagged complementing gene and its ~1 Kbp 5' UTR region was placed next to the bacterial cytosine deaminase (CD) gene (flanked by the 5' and 3' dhfr upstream regions [105]), and both genes were flanked by 5' and 3' OMPDC locus targeting flanks for insertion of the complementing gene and the CD marker at the OMPDC locus. Complemented strains were selected in medium containing mycophenolic acid (MPA) + xanthine (X) + cytosine and genotypes were verified using PCR 4 and PCR 5 as shown. (TIF) [file pgen.1006189.s007.tif]

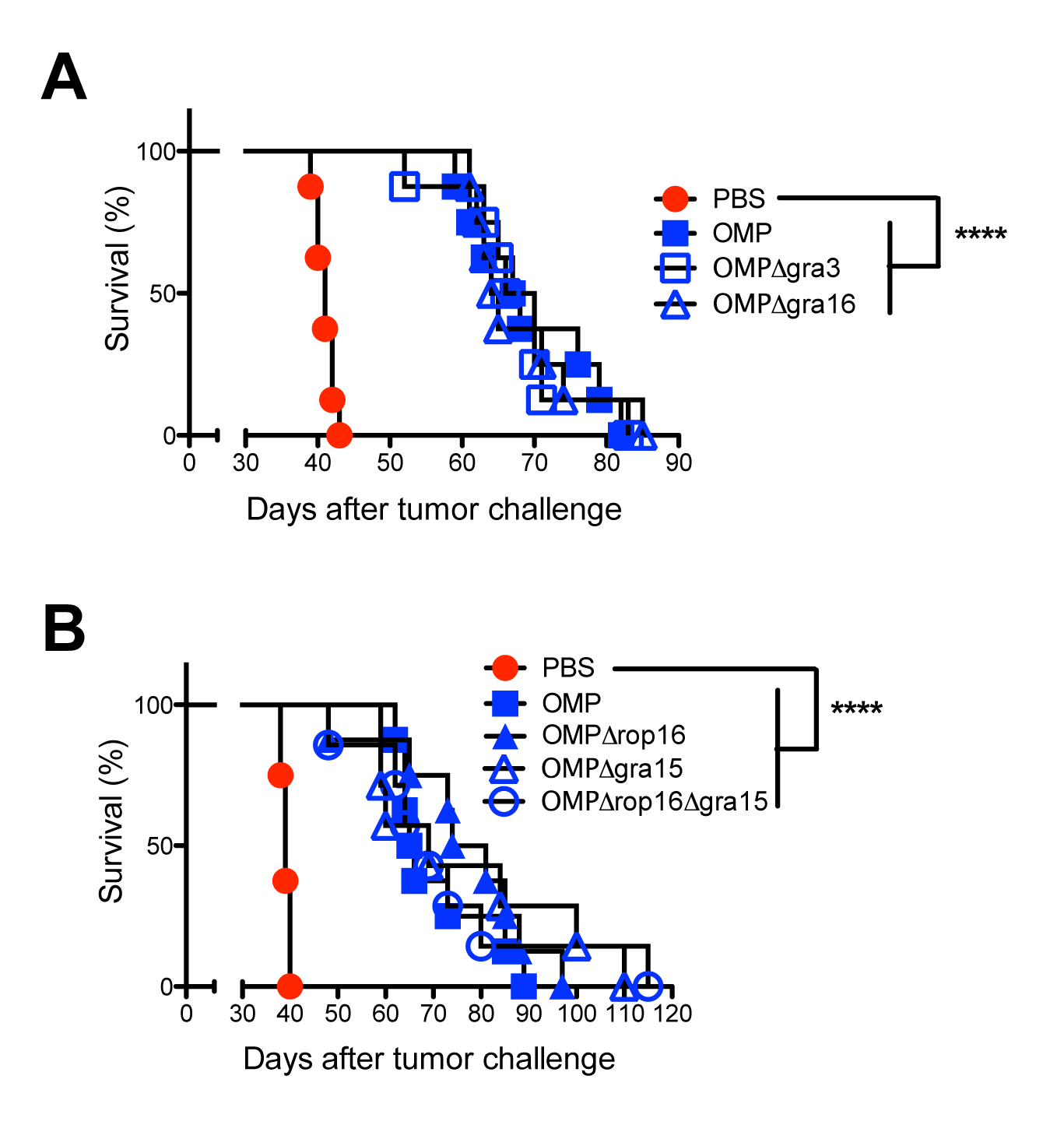

Supplement: S8 Fig — ID8DV ovarian tumors were established in C57BL/6 mice and groups of mice were treated with PBS, or were vaccinated i.p. with tachyzoites of uracil auxotrophs, or (A) were vaccinated i.p. with OMP or OMP lacking GRA proteins GRA3 or GRA16, or (B) vaccinated i.p. with OMP or OMP lacking GRA15 or ROP16, or GRA15 and ROP16. Data is representative of two independent experiments. ****P<0.0001. (TIF) [file pgen.1006189.s008.tif]

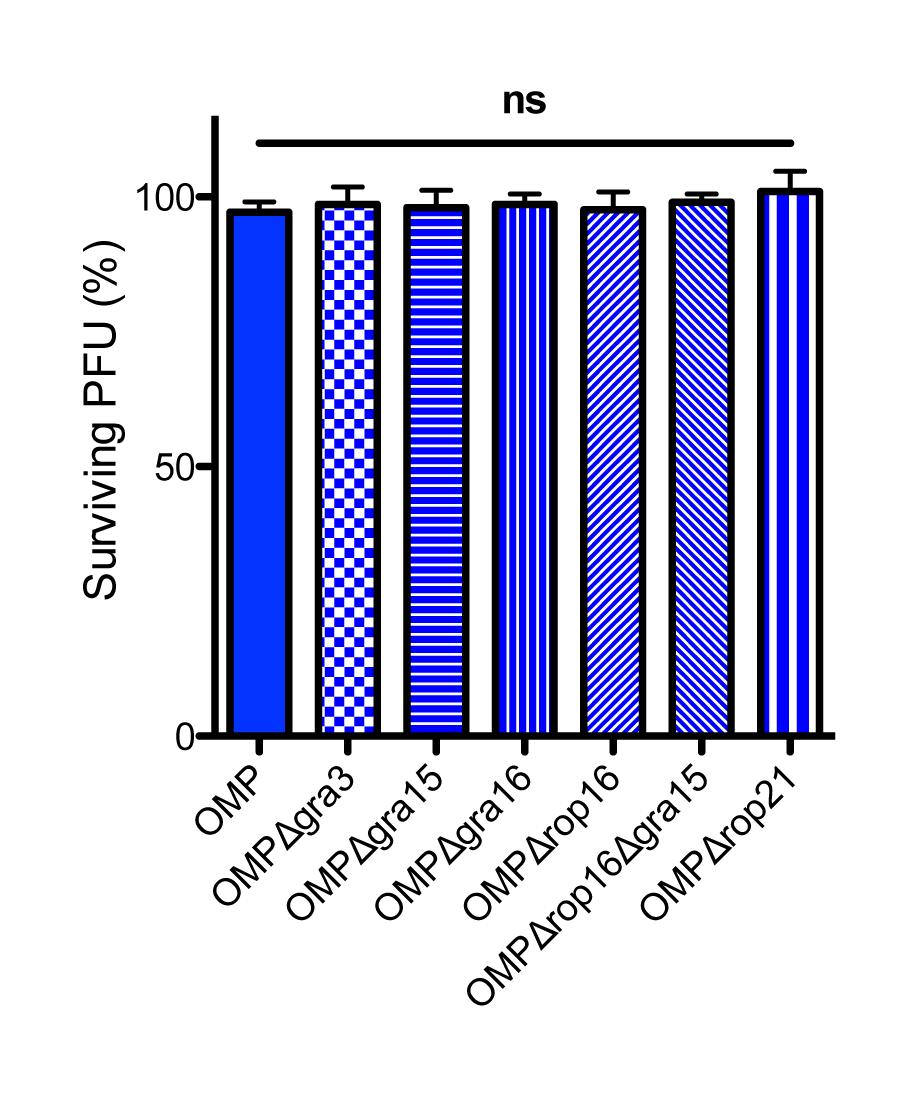

Supplement: S9 Fig — PFU survival was measured in IFN-γ activated MEFs infected with uracil auxotrophic vaccine mutants that did not affect the antitumor response. ns was not significant (TIF) [file pgen.1006189.s009.tif]

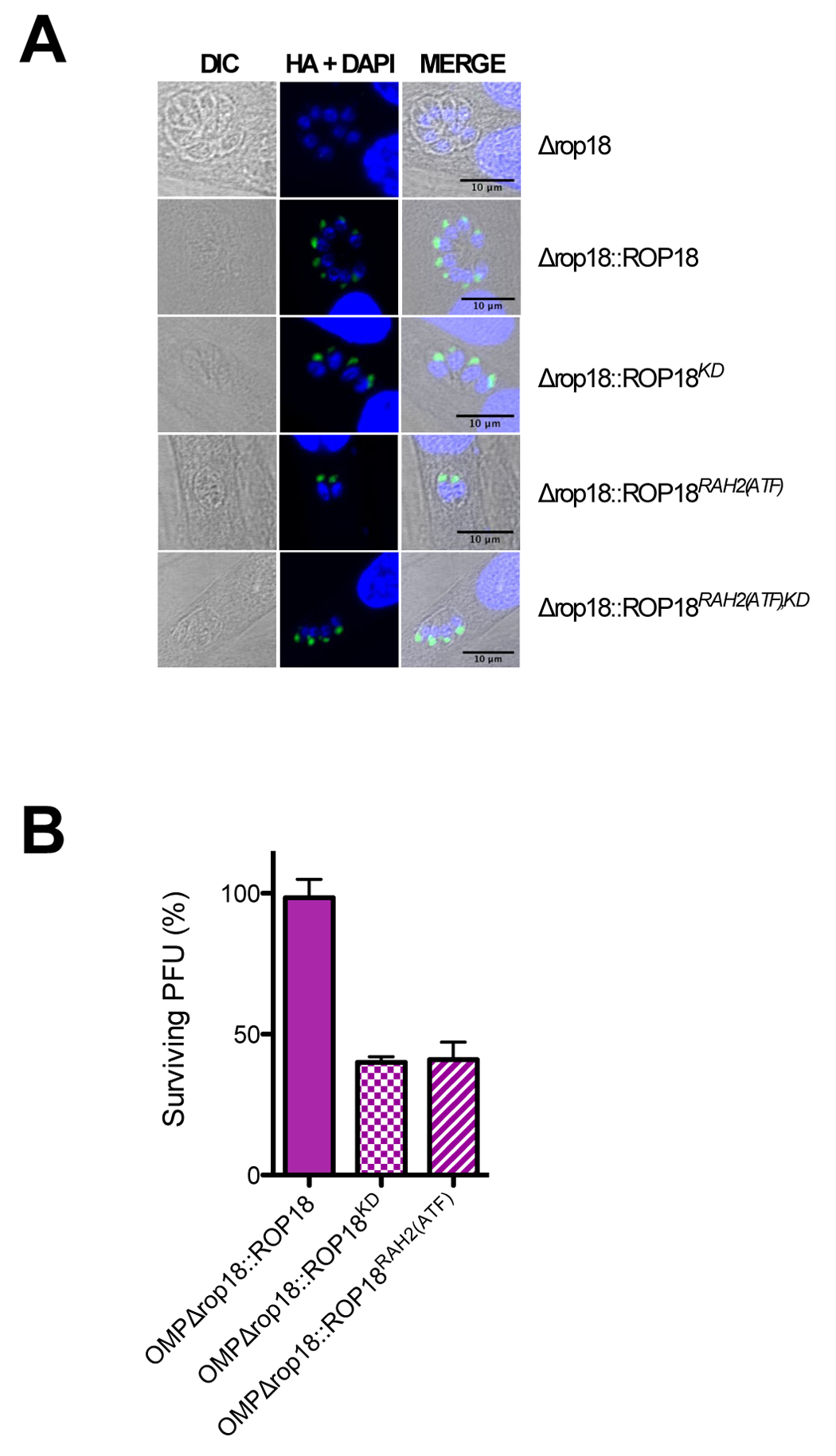

Supplement: S10 Fig — (A) Rhoptry localization of expressed ROP18 gene alleles. Immunofluorescence validation of apical rhoptry localization of expressed C-terminal HA-tagged ROP18 alleles in complemented OMPΔrop18 strains: wild-type ROP18 (OMPΔrop18::ROP18), a kinase-dead (KD) ROP18 (OMPΔrop18::ROP18KD), a RAH2 domain deleted ROP18 (OMPΔrop18::ROP18RAH2(ATF)), or a kinase-dead and RAH2(ATF) mutant (OMPΔrop18::ROP18 RAH2(ATF),KD). DAPI stains the nuclei of both parasites and the host cells they invaded. The HA tag (revealed) by green fluorescence associated with apical rhoptry organelles is present in the complemented ROP18 strains and is absence in the ROP18 deleted strain. Vacuole locations are shown by differential interference contrast (DIC) microscopy. (B) Bone marrow derived macrophages were stimulated with IFN-γ and TNF-α and parasite survival (measured as PFU) was determined for complemented OMPΔrop18 strains: wild-type ROP18 (OMPΔrop18::ROP18), a kinase-dead (KD) ROP18 (OMPΔrop18::ROP18KD), or a RAH2 domain deleted ROP18 (OMPΔrop18::ROP18RAH2(ATF)). (TIF) [file pgen.1006189.s010.tif]
