# Supplementary material for: Secretion of Rhoptry and Dense Granule Effector Proteins by Nonreplicating Toxoplasma gondii Uracil Auxotrophs Controls the Development of Antitumor Immunity
Source: PLoS Genet. 2016 Jul 22;12(7):e1006189. doi: 10.1371/journal.pgen.1006189 (PMC4957766; doi:10.1371/journal.pgen.1006189)
Supplement: S1 Table — Primer pairs used to create 5' and 3' gene targeting flanks. The chromosomal location of each gene and nucleotides targeted for deletion are shown for each plasmid construct. (DOCX) [file pgen.1006189.s011.docx]

**S1 Table. Oligonucleotide primers used for development of GRA or ROP knockout plasmids.**

**Primer Name and Sequence Primer Use KO Construct Corresponding ToxoDB locus KO**

**PMiniHXF GATAAGCTTGATCAGCACGAAACCTTG** HXGPRT cassette forward primer HXGPRT mini cassette

**PMiniHXR CCGCTCTAGAACTAGTGGATCCC** HXGPRT cassette reverse primer

**GRA2TF1** *TTGGGTAACGCCAGGGTTTTCCCAGTCACGACG*GTTTAAAC**CTATGGAACAAGCCGTGTGTCGATAG** RH GRA2 KO 5’ forward primer pRS416.GRA2T TGGT1_227620 chrX 779,985 to 780,855 (-)

**GRA2TR1** *GCGGGTTTGAATGCAAGGTTTCGTGCTGATCAA*ACTAGT**TCGCACTTAGCCTAAGGGACGAC** RH GRA2 KO 5’ reverse primer

**GRA2TF2** *TTCTGGCAGGCTACAGTGACACCGCGGTGGAGG*ACTAGT**TGGAGAGTGACAGAAGCACTACGAC** RH GRA2 KO 3’ forward primer

**GRA2TR2** *GTGAGCGGATAACAATTTCACACAGGAAACAGC*GCGGCCGC**CGCCCTCGTGATTCCTCCAAG** RH GRA2 KO 3’ reverse primer

**GRA3TF1** *TTGGGTAACGCCAGGGTTTTCCCAGTCACGACG*GTTTAAAC**CATAACAACCCAGGTCTCGCGTC** RH GRA3 KO 5’ forward primer pRS416.GRA3T TGGT1_227280 chrX 956,328 to 957,082 (-)

**GRA3TR1** *GCGGGTTTGAATGCAAGGTTTCGTGCTGATCAA*ACTAGT**GTGCAGGTGTCCCACTGTCG** RH GRA3 KO 5’ reverse primer

**GRA3TF2** *TTCTGGCAGGCTACAGTGACACCGCGGTGGAGG*ACTAGT**GAGGCAACCCTTCATGAGTTCGG** RH GRA3 KO 3’ forward primer

**GRA3TR2** *GTGAGCGGATAACAATTTCACACAGGAAACAGC*CTCGAG**CGAGCAGCGATGTAGTTCACGATC** RH GRA3 KO 3’ reverse primer

**GRA12TF1** *TTGGGTAACGCCAGGGTTTTCCCAGTCACGACG*GTTTAAAC**CCGACGACATCTTGGTCACACC** RH GRA12 KO 5’ forward primer pRS416.GRA12T TGGT1_288650 chrIX 2,468,014 to 2,469,809 (-)

**GRA12TR1** *GCGGGTTTGAATGCAAGGTTTCGTGCTGATCAA*ACTAGT**ACTGGCAGGCACTCGATAGGG** RH GRA12 KO 5’ reverse primer

**GRA12TF2** *TTCTGGCAGGCTACAGTGACACCGCGGTGGAGG*ACTAGT**GTTGGAGCAGCTCTTGCTCGAG** RH GRA12 KO 3’ forward primer

**GRA12TR2** *GTGAGCGGATAACAATTTCACACAGGAAACAGCGCGGCCGC****CCATCTCCCATTGTTGAAAGAGTGCG*** RH GRA12 KO 3’ reverse primer

**GRA15TF1A** *TTGGGTAACGCCAGGGTTTTCCCAGTCACGACG*GTTTAAAC**GATGCCTCTAACACGCGTATGGTG** RH GRA15 KO 5’ forward primer pRS416.GRA15T TGGT1_275470 chrX 7,172,374 to 7,176,692 (+)

**GRA15TR1A** *GCGGGTTTGAATGCAAGGTTTCGTGCTGATCAAGCGGCCGC***CAGGGAAGTAGCAGGCATGAAGC** RH GRA15 KO 5’ reverse primer

**GRA15TF2** *TTCTGGCAGGCTACAGTGACACCGCGGTGGAGGGCGGCCGC***GAAACCACCGATCCAGTGGACTC** RH GRA15 KO 3’ forward primer

**GRA15PR2** *GTGAGCGGATAACAATTTCACACAGGAAACAGC*ACTAGT**AGTCCACGTGGTGGTCAGACC** RH GRA15 KO 3’ reverse primer

**GRA16TF1** *TTGGGTAACGCCAGGGTTTTCCCAGTCACGACG*GTTTAAAC**CAGGCGGTTCACATCGGAAGTG** RH GRA16 KO 5’ forward primer pRS416.GRA16T TGGT1_208830 chr1b 904,590 to 906,458 (-)

**GRA16TR1** *GCGGGTTTGAATGCAAGGTTTCGTGCTGATCAA*AGATCT**CGGATTGGATGTCCTCGCTCAG** RH GRA16 KO 5’ reverse primer

**GRA16TF2** *TTCTGGCAGGCTACAGTGACACCGCGGTGGAGG*AGATCT**AGGCGAGTCATACTGAGCCGAC** RH GRA16 KO 3’ forward primer

**GRA16TR2** *GTGAGCGGATAACAATTTCACACAGGAAACAGC*GCGGCCGC**TCCGTTCTGACGAGGTCAGTGC** RH GRA16 KO 3’ reverse primer

**GRA24TF1** *TTGGGTAACGCCAGGGTTTTCCCAGTCACGACG*GTTTAAAC**ACAGATGTCCTCATCTCAGCGTCC** RH GRA24 KO 5’ forward primer pRS416.GRA24T TGGT1_230180 chrVIII 662,193 to 666,904 (-)

**GRA24TR1** *GCGGGTTTGAATGCAAGGTTTCGTGCTGATCAA*CTCGAG**GCTGGACCGCTCTCATCAACC** RH GRA24 KO 5’ reverse primer

**GRA24F2** *TTCTGGCAGGCTACAGTGACACCGCGGTGGAGG*CTCGAG**GCCCTATTGGTACTGGCAAAGCC** RH GRA24 KO 3’ forward primer

**GRA24R2** *GTGAGCGGATAACAATTTCACACAGGAAACAGC*GCGGCCGC**ACTGCGCTGATACCCGTCGTG** RH GRA24 KO 3’ reverse primer

**ROP5TF1** *TTGGGTAACGCCAGGGTTTTCCCAGTCACGACG*GTTTAAAC**GAGAAGATACTGATGTGCTGCACACG** RH ROP5 KO 5’ forward primer pRS426.GRA5T TGME49_308090 chrXII 564,553 to 577,981 (-)

**ROP5TR1** *GCGGGTTTGAATGCAAGGTTTCGTGCTGATCAA*ACTAGT**GGCATTGCAGAATCTATGCAGCCAG** RH ROP5 KO 5’ reverse primer

**ROP5TF2** *TTCTGGCAGGCTACAGTGACACCGCGGTGGAGG*ACTAGT**TCCACTCACTGGTGTAGTCGATGC** RH ROP5 KO 3’ forward primer

**ROP5TR2** *GTGAGCGGATAACAATTTCACACAGGAAACAGC*GCGGCCGC**GTGTAGCGTGCCACACTTCGC** RH ROP5 KO 3’ reverse primer

**ROP16TF1** *TTGGGTAACGCCAGGGTTTTCCCAGTCACGACG*GTTTAAAC**GGCGTTCTGTGTTAGCTGCCAG** RH ROP16 KO 5’ forward primer pRS416.ROP16T TGGT1_262730 chrVIIb 1,028,631 to 1,030,829 (-)

**ROP16TR1** *GCGGGTTTGAATGCAAGGTTTCGTGCTGATCAA*ACTAGT**CATAGGCACTACCAGTGGTGCATTG** RH ROP16 KO 5’ reverse primer

**ROP16TF2** *TTCTGGCAGGCTACAGTGACACCGCGGTGGAGG*ACTAGT**CGAATCTGATCCAGCAGTGATGGG** RH ROP16 KO 3’ forward primer

**ROP16TR2** *GTGAGCGGATAACAATTTCACACAGGAAACAGC*GCGGCCGC**CCCATGTCTCTTAAGGTGTGCGTC** RH ROP16 KO 3’ reverse primer

**ROP17TF1** *TTGGGTAACGCCAGGGTTTTCCCAGTCACGACG*GTTTAAAC**GCCGGACTGTAACCCGAAGC** RH ROP17 KO 5’ forward primer pRS416.ROP17T TGGT1_258580 chrVIIb 3,286,893 to 3,289,662 (-)

**ROP17TR1** *GCGGGTTTGAATGCAAGGTTTCGTGCTGATCAA*GCGGCCGC**AGACTGGTGCAGCTGGCCTG** RH ROP17 KO 5’ reverse primer

**ROP17TF2** *TTCTGGCAGGCTACAGTGACACCGCGGTGGAGG*GCGGCCGC**GACTACGCCACATGTACCACTCG** RH ROP17 KO 3’ forward primer

**ROP17TR2** *GTGAGCGGATAACAATTTCACACAGGAAACAGC*ACTAGT**AGGCGACTCCGTCAGTCTTCC** RH ROP17 KO 3’ reverse primer

**ROP18TF1** *TTGGGTAACGCCAGGGTTTTCCCAGTCACGACG*GTTTAAAC**GGAACTCTGGTTGAACCTGCGTG** RH ROP18 KO 5’ forward primer pRS416.ROP18T TGGT1_ 205250 chrVIIa 1,426,891 to 1,428,633 (-)

**ROP18TR1** *GCGGGTTTGAATGCAAGGTTTCGTGCTGATCAA*TCTAGA**AGAGGTGCATAGCGTGGCTAGC** RH ROP18 KO 5’ reverse primer

**ROP18TF2** *TTCTGGCAGGCTACAGTGACACCGCGGTGGAGG*TCTAGA**GATGCTCCCGCTACAAGCCTTG** RH ROP18 KO 3’ forward primer

**ROP18TR2** *GTGAGCGGATAACAATTTCACACAGGAAACAGC*GCGGCCGC**CGCAGAGTCGATACGAACAACATGG** RH ROP18 KO 3’ reverse primer

**ROP21TF1** *TTGGGTAACGCCAGGGTTTTCCCAGTCACGACG*ACTAGT**GTCACACCAAGCACTGGGATGC** RH ROP21 KO 5’ forward primer pRS416.ROP21T TGGT1_263220 chrVIIb 673,993 to 679,112 (+)

**ROP21TR1** *GCGGGTTTGAATGCAAGGTTTCGTGCTGATCAA*GTTTAAAC**CAACACTGCGGCGACTTACGG** RH ROP21 KO 5’ reverse primer

**ROP21TF2** *TTCTGGCAGGCTACAGTGACACCGCGGTGGAGG*GTTTAAAC**GCAAGCAGGCAGCGACATCG** RH ROP21 KO 3’ forward primer

**ROP21TR2** *GTGAGCGGATAACAATTTCACACAGGAAACAGC*GCGGCCGC**TCCCAAACGTGTTCCAATAGTCCG** RH ROP21 KO 3’ reverse primer

**ROP35TF1** *TTGGGTAACGCCAGGGTTTTCCCAGTCACGACG*GTTTAAAC**CCTCCTACACAGGCAATGTCCG** RH ROP35 KO 5’ forward primer pRS416.ROP35T TGGT1_304740 chrVIIa 656,106 to 653,233 (-)

**ROP35TR1** *GCGGGTTTGAATGCAAGGTTTCGTGCTGATCAA*ACTAGT**CTGGATGTCACACTCGACTCCG** RH ROP35 KO 5’ reverse primer

**ROP35TF2** *TTCTGGCAGGCTACAGTGACACCGCGGTGGAGG*ACTAGT**GCTGAAACAGGTCATGGAAGACCC** RH ROP35 KO 3’ forward primer

**ROP35TR2** *GTGAGCGGATAACAATTTCACACAGGAAACAGC*GCGGCCGC**CACGCTGTGTCGCAGACTCG** RH ROP35 KO 3’ reverse primer

**ROP38TF1** *TTGGGTAACGCCAGGGTTTTCCCAGTCACGACG*GTTTAAAC**GGTGGTCTGAGAGTTTGATCACGG** RH ROP38 KO 5’ forward primer pRS416.ROP38T TGME49_242110 chrVI 1,892,051 to 1,923,747 (+)

**ROP38TR1** *GCGGGTTTGAATGCAAGGTTTCGTGCTGATCAA*ACTAGT**AATTGTGGGGAGTCCGCTGGTG** RH ROP38 KO 5’ reverse primer TGGT1_242100 chrVI 1,829,335 to? not in database (+)

**ROP38TF2** *TTCTGGCAGGCTACAGTGACACCGCGGTGGAGG*ACTAGT**TCGCTAACTGGGTAGCCTGCTG** RH ROP38 KO 3’ forward primer

**ROP38TR2** *GTGAGCGGATAACAATTTCACACAGGAAACAGC*GCGGCCGC**GCGACTGTCTTGCCTACCACTC** RH ROP38 KO 3’ reverse primer

**ROP5PF1** *TTGGGTAACGCCAGGGTTTTCCCAGTCACGACG*GTTTAAAC**GAGAAGATACTGATGTGCTGCACACG** Pru ROP5 KO 5’ forward primer pRS416.ROP5P TGME49_308090 chrXII 577,981 to 564,554 (-)

**ROP5PR1** *GCGGGTTTGAATGCAAGGTTTCGTGCTGATCAA*ACTAGT**GGCATTGCAGAATCTATGCAGCCAG** Pru ROP5 KO 5’ reverse primer

**ROP5PF2** *TTCTGGCAGGCTACAGTGACACCGCGGTGGAGG*ACTAGT**TCCACTCACTGGTGTAGTCGATGC** Pru ROP5 KO 3’ forward primer

**ROP5PR2** *GTGAGCGGATAACAATTTCACACAGGAAACAGC*GCGGCCGC**GTGTAGCGTGCCACACTTCGC** Pru ROP5 KO 3’ reverse primer

**ROP18PF1** *TTGGGTAACGCCAGGGTTTTCCCAGTCACGACG*GTTTAAAC**GGAACTCTGATTGAACCTGCGTG** Pru ROP18 KO 5’ forward primer pRS416.ROP18P TGME49_205250 chrVIIa 1,516,300 to 1,514,558 (-)

**ROP18PR1** *GCGGGTTTGAATGCAAGGTTTCGTGCTGATCAA*TCTAGA**AGAGGTGCATAGCGTGGCTAGC** Pru ROP18 KO 5’ reverse primer

**ROP18PF2** *TTCTGGCAGGCTACAGTGACACCGCGGTGGAGG*TCTAGA**GATGCTCCCGCTACAAGCCTTG** Pru ROP18 KO 3’ forward primer

**ROP18PR2** *GTGAGCGGATAACAATTTCACACAGGAAACAGC*GCGGCCGC**TGCAGAGTCGATACGAACAACATGG** Pru ROP18 KO 3’ reverse primer

*Italicised nucleotides indicate regions of crossover in yeast recombination cloning, underlined nucleotides indicate restriction enzyme sites, and bold nucleotides indicate specific priming target regions (ToxoDB version 26.0). Note# ROP5 and ROP38 loci are addressed to the corresponding ME49 strain loci as these loci for the GT1 strain are not yet listed in ToxoDB version 26.0.
