# Supplementary material for: Secretion of Rhoptry and Dense Granule Effector Proteins by Nonreplicating Toxoplasma gondii Uracil Auxotrophs Controls the Development of Antitumor Immunity
Source: PLoS Genet. 2016 Jul 22;12(7):e1006189. doi: 10.1371/journal.pgen.1006189 (PMC4957766; doi:10.1371/journal.pgen.1006189)
Supplement: S2 Table — (DOCX) [file pgen.1006189.s012.docx]

**Primer name Sequence Primer use**

**S2 Table. Oligonucleotide primers used for validation of Δrop and Δgra knockouts.**

**5’DHFRCXR**  **ACTGCGAACAGCAGCAAGATCG 5' reverse primer for validation of HX and 5' flank integration**

**3’DHFRCXF GTTGGCCTACGTGACTTGCTGATG 3' forward primer for validation of HX and 3' flank integration**

**GRA2TCXF GTTAACAGTTCCCTTGTGGCTGGTC forward primer for validation of 5' gra2 flank integration**

**GRA2TDF2 GCTTCTTCTCCACATATCGCCTCAC forward gra2 deletion validation primer**

**GRA2TDR2 GGGAGTGGTGGTGTATGTTCACC reverse gra2 deletion validation primer**

**GRA2TCXR CCCCAGGATAATGCAGAAAAGCTGG reverse primer for validation of 3' gra2 flank integration**

**GRA3TCXF GCAAGGTCAACAGGGGTGCTTC forward primer for validation of 5' gra3 flank integration**

**GRA3TDF AATCAATCAGGCTCTTGCAAGAACCAG forward gra3 deletion validation primer**

**GRA3TDR2 GTCATCACTCGACGACGATAGGTAAG reverse gra3 deletion validation primer**

**GRA3TCXR TCCGGACGTGTGTCCTGAGAG reverse primer for validation of 3' gra3 flank integration**

**GRA12TCXF2 CTTCTGGTTCGGGCCAGCAC forward primer for validation of 5' gra12 flank integration**

**GRA12TDF CGATTTCGGGTGTACTTGTCAGCG forward gra12 deletion validation primer**

**GRA12TDR ATGGAAGACCGTTTCTCTACAGGCTG reverse gra12 deletion validation primer**

**GRA12TCXR AGAGTTGAGTGCCTACGTCCCTC reverse primer for validation of 3' gra12 flank integration**

**GRA15TCXF GCCACGTGTAGTATCCTCGTTGC forward primer for validation of 5' gra15 flank integration**

**GRA15TDF GTGCGCATTTGGGTGCTGTCC forward gra15 deletion validation primer**

**GRA15TDR CACGAACACCCCTTCAGACAAGC reverse gra15 deletion validation primer**

**GRA15TCXR AGGCCCAAACTGGATCGAGGG reverse primer for validation of 3' gra15 flank integration**

**GRA16TCXF CCTGTCCGTGTAACCAGCAAACC forward primer for validation of 5' gra16 flank integration**

**GRA16TDF CTCTGGTATTCACCGGTTCGTCG forward gra16 deletion validation primer**

**GRA16TDR CTTCGAGTGGATCCGAATCCCATG reverse gra16 deletion validation primer**

**GRA16TCXR GCTTGGAGGTCAGCATGTCCAAC reverse primer for validation of 3' gra16 flank integration**

**GRA24TCXF GCCGAAGCTGTTTACGGCAGAC forward primer for validation of 5' gra24 flank integration**

**GRA24TDF CAGCCAACAACGACACTCAGCG forward gra24 deletion validation primer**

**GRA24TDR GTTGGCCTACCATGTGCTGAACC reverse gra24 deletion validation primer**

**GRA24TCXR CAGGCAACGCCGTGATCCAC reverse primer for validation of 3' gra24 flank integration**

**ROP5TCXF GAATGAGAGCACGCGAAGCTGC forward primer for validation of RH and Pru 5' rop5 flank integration**

**ROP5TDF CCTGCAGCGATGGTTGAGGC forward RH and Pru rop5 deletion validation primer**

**ROP5TDR TGTCCTGGACTCAGCTGAGCG reverse RH and Pru rop5 deletion validation primer**

**ROP5TCXR GCGAACGCGTACTTGTACGCTG reverse primer for validation of RH and Pru 3' rop5 flank integration**

**ROP16TCXF GCAACTACTTCCGACGGAACCGTC forward primer for validation of 5' rop16 flank integration**

**ROP16TDF TCCTGGTCAGAGCGCTACATTGG forward rop16 deletion validation primer**

**ROP16TDR GGAAACACTTCGTCAACAGCTGACTC reverse rop16 deletion validation primer**

**ROP16TCXR CCTTAACAGGCAAATGAACACGAGCT reverse primer for validation of 3' rop16 flank integration**

**ROP16TCLFA CAGCTTCATGGTGACAGTTCTGATGC forward validation primer for removal of HX from rop16**

**ROP17TCXF GCTGGAATTGACGCATCGCTCG forward primer for validation of 5' gra2 flank integration**

**ROP17TDF TGCGTTCGTGGTGAGCTAGGAC forward rop17 deletion validation primer**

**ROP17TDR GACAGCGTATGGCATCTATGCCAC reverse rop17 deletion validation primer**

**ROP17TCXR CAGTGAGATGGCAGGTTGCCAG reverse primer for validation of 3' rop17 flank integration**

**ROP18TCXF TGGCTGTGGTGAGGCACTCG forward primer for validation of 5' rop18 flank integration**

**ROP18TDF2 GAGCGACAGAAAGCACTCGAGAC forward rop18 deletion validation primer**

**ROP18TDR2 TGCCGATGACGCATCCAGAATTCC reverse rop18 deletion validation primer**

**ROP18PCXR2 TGGTTCCGGATACTGAGAGACACTAG reverse primer for validation of 3' rop18 flank integration**

**ROP18PCXF GTGGCTGTGGTGAGGCACTC forward primer for validation of Pru 5' rop18 flank integration**

**ROP18PDF2 GAGCGACAGAAAGCACTCGAGAC forward Pru rop18 deletion validation primer**

**ROP18PDR2 GGAATTCTGGATGCGTCATCGGCA reverse Pru rop18 deletion validation primer**

**ROP18PCXR2 TGGTTCCGGATACTGAGAGACACTAG reverse primer for validation of 3' Pru rop18 flank integration**

**ROP21TCXF ACCGGAGAATACGCTCTCCGC forward primer for validation of 5' rop21 flank integration**

**ROP21TDF CCAGCGTTGACAGCATCCAGC forward rop21 deletion validation primer**

**ROP21TDR GCTCACCTTCTCGGCAGTCCA reverse rop21 deletion validation primer**

**ROP21TCXR AGCGATTCCTCTCTGTATGTGCTCC reverse primer for validation of 3' rop21 flank integration**

**ROP35TCXF CGCTGATTCGTCGTGCCTGC forward primer for validation of 5' rop35 flank integration**

**ROP35TDF GACGCAGACGTTACAGTGTTCAGG forward rop35 deletion validation primer**

**ROP35TDR GTCACGAACGCGAGAAACACCC reverse rop35 deletion validation primer**

**ROP35TCXR CACGGGAAAGCAATGACGGAGC reverse primer for validation of 3' rop35 flank integration**

**ROP38TCXF GGTGAGCGCTATGGCGTTGC forward primer for validation of 5' rop38 flank integration**

**ROP38TDF CGTCAGGCAACGTGGTTGTACG forward rop38 deletion validation primer**

**ROP38TDR ATGTTGCGGGTCCCTGTAGCG reverse rop38 deletion validation primer**

**ROP38CXR GCGCTCTTGCCGAAGTGTCG reverse primer for validation of 3' rop38 flank integration**
