# Supplementary material for: Secretion of Rhoptry and Dense Granule Effector Proteins by Nonreplicating Toxoplasma gondii Uracil Auxotrophs Controls the Development of Antitumor Immunity
Source: PLoS Genet. 2016 Jul 22;12(7):e1006189. doi: 10.1371/journal.pgen.1006189 (PMC4957766; doi:10.1371/journal.pgen.1006189)
Supplement: S3 Table — Complementing genes were C-terminally HA tagged and were constructed using one or two PCR segments as shown. (DOCX) [file pgen.1006189.s013.docx]

**S3 Table. Oligonucleotide primers used for construction of complementation targeting plasmids.**

**Primer Name and Sequence Primer Use Construct Corresponding ToxoDB locus KO**

**PMiniHXF GATAAGCTTGATCAGCACGAAACCTTG** CD cassette forward primer CD mini cassette

**PMiniHXR CCGCTCTAGAACTAGTGGATCCC** CD cassette reverse primer

**OMPFXF1**  *TTGGGTAACGCCAGGGTTTTCCCAGTCACGACG*GTTTAAAC**GCCGTAGTGTACCCGATGATGC** Ompdc utr forward primer All 5' Omp target flanks TGGT1_259690 chrVIIb 2,683,496 to 2,686,820 (-)

**OMPFXR1 CCGAATAGCAGTGTTGGACACGTG** Ompdc 5utr reverse primer

**OMPXF2 CD** *TTCTGGCAGGCTACAGTGACACCGCGGTGGAGG*GCGGCCGC**CGATGACGGCGAAGTTGACTG** Ompdc 3utr forward primer All 3' Omp target flanks

**OMPXR2** *GTGAGCGGATAACAATTTCACACAGGAAACAGC*GCTGAGC**CGGTTGACGAATAGTCTTCGCTGC** Ompdc 3utr reverse primer

**OROP18XF1A** *GGTACGTTGCACGTGTCCAACACTGCTATTCGGGCATG***CGCGGAAGTAACTCGAGTCGATGC** ROP18 5' forward primer pRS416.OROP18CD TGGT1_205250 chrVIIa 1,426,785 to 1,429,090 (-)

**OROP18XR1AHACD** *GCGGGTTTGAATGCAAGGTTTCGTGCTGATCAATCATCGGG*CGGCCGCTCTAGATCAAAGAG ROP18 3' reverse primer

CGTAATCTGGAACATCGTATGGGTA**TTCTGTGTGGAGATGTTCCTGCTGTTC**

**OROP18XF1A** *GGTACGTTGCACGTGTCCAACACTGCTATTCGGGCATG***CGCGGAAGTAACTCGAGTCGATGC**  ROP18Δ(ATF) 5’ forward primer pRS416.OROP18ΔATFCD TGGT1_205250 chrVIIa delta AA147-164 **ROP18TΔATFR1** **CTTGAGGAAAGTACCTCCGGATTCCGGACAGACGGGGTCTCGAGGAGCTA** ROP18Δ(ATF) 5' reverse primer

**ROP18TΔATFF1 TAGCTCCTCGAGACCCCGTCTGTCCGGAATCCGGAGGTACTTTCCTCAAG**  ROP18Δ(ATF) 3' forward primer

**OROP18XR1AHACD** *GCGGGTTTGAATGCAAGGTTTCGTGCTGATCAATCATCGGG*CGGCCGCTCTAGA ROP18Δ(ATF) 3’ reverse primer

TCAAAGAGCGTAATCTGGAACATCGTATGGGTA**TTCTGTGTGGAGATGTTCCTGCTGTTC**

**OROP18XF1A** *GGTACGTTGCACGTGTCCAACACTGCTATTCGGGCATG***CGCGGAAGTAACTCGAGTCGATGC** ROP18 5’ forward primer pRS416.OROP18KDCD TGGT1_205250 chrVIIa kinase dead D409A

**ROP18TKIND/A R** **TTCGCCGGTTTGATAGCCGTATGCACAATTCCCTGAGC** ROP18 5’ reverse primer

**ROP18TKIND/A F GCTCAGGGAATTGTGCATACGGCTATCAAACCGGCGAA**  ROP18 3’ forward primer

**OROP18XR1AHACD** *GCGGGTTTGAATGCAAGGTTTCGTGCTGATCAATCATCGGG*CGGCCGCTCTAGATCA ROP18 3’ reverse primer

AAGAGCGTAATCTGGAACATCGTATGGGTA**TTCTGTGTGGAGATGTTCCTGCTGTTC**

**OROP18XF1A** *GGTACGTTGCACGTGTCCAACACTGCTATTCGGGCATG***CGCGGAAGTAACTCGAGTCGATGC**  ROP18Δ(ATF),KD 5’ forward primer pRS416.OROP18ΔATFKDCD TGGT1_205250 chrVIIa delta AA147-164 **ROP18TΔATFR1** **CTTGAGGAAAGTACCTCCGGATTCCGGACAGACGGGGTCTCGAGGAGCTA** ROP18Δ(ATF),KD 5' reverse primer kinase dead D409A

**ROP18TΔATFF1 TAGCTCCTCGAGACCCCGTCTGTCCGGAATCCGGAGGTACTTTCCTCAAG**  ROP18Δ(ATF),KD 3' forward primer

**ROP18TKIND/A R** **TTCGCCGGTTTGATAGCCGTATGCACAATTCCCTGAGC** ROP18Δ(ATF),KD 5' reverse primer

**ROP18TKIND/A F GCTCAGGGAATTGTGCATACGGCTATCAAACCGGCGAA**  ROP18Δ(ATF),KD 3’ forward primer

**OROP18XR1AHACD** *GCGGGTTTGAATGCAAGGTTTCGTGCTGATCAATCATCGGG*CGGCCGCTCTAGA ROP18Δ(ATF),KD 3’ reverse primer

TCAAAGAGCGTAATCTGGAACATCGTATGGGTA**TTCTGTGTGGAGATGTTCCTGCTGTTC**

**OPROP35F1** *GGTACGTTGCACGTGTCCAACACTGCTATTCGG*ACTAGT**TCCAAAGGTCTCCGCACCCAG**  ROP35 5’ forward primer pRS416.OROP353XHACD TGGT1_304740 chrVIIa 6,216,171 to 6,220,719 (-) **OPROP35R1** ***GAGATCGCTCGAGACACAACTGC***  ROP35 5’ reverse primer

**OROP35FA1REC *GTGCATGCATTCAAGCAGTTGTGTC***  ROP35 3' forward primer

**OROP353XHARKPN** *GCGGGTTTGAATGCAAGGTTTCGTGCTGATCAA*GGTACCTCTAGACTAAACAGCGTAA ROP35 3' reverse primer

TCTGGAACGTCATATGGATAGGATCCTGCATAGTCCGGGACGTCATAGGGATAGCCAG

CGTAATCTGGAACATCGTATGGGTAGGCGCC**TTCGTTTTCCTGTTCATGGTCTTGTTCC**

*Italicised nucleotides indicate regions of crossover in yeast recombination cloning, underlined nucleotides indicate restriction enzyme sites, and bold nucleotides indicate specific genomic priming target regions (ToxoDB version 26.0). Note# ROP5 and ROP38 loci are addressed to the corresponding ME49 loci as these loci for the GT1 strain are not yet listed in ToxoDB version 26.0.
