# Supplementary material for: Secretion of Rhoptry and Dense Granule Effector Proteins by Nonreplicating Toxoplasma gondii Uracil Auxotrophs Controls the Development of Antitumor Immunity
Source: PLoS Genet. 2016 Jul 22;12(7):e1006189. doi: 10.1371/journal.pgen.1006189 (PMC4957766; doi:10.1371/journal.pgen.1006189)
Supplement: S4 Table — (DOCX) [file pgen.1006189.s014.docx]

**Primer Sequence Primer use**

**S4 Table. Oligonucleotide primers used to validate insertion of complementing genes at the *OMPDC* locus.**

**5’OMCXF CAGCAGAGCAATACGGAGGCTGT 5' FP for validation of all 5' flank integrations of ΔGOI Complementation Constructs at OMPDC**

**3’OMCXR CACTCGCTAAAACAGCAACGGTTGAC 3' RP for validation of all 3' flank integrations of ΔGOI Complementation Constructs at OMPDC**

**CODDF CGTTGCTGGAAGAGGCGTTACG 3' FP for validation of all 3' flank integrations of ΔGOI Complementation Constructs**

**RP18COMPXRT GAGCCATTTGACTGCAGTGTCTCG 5' RP for validation of all 5' flank integrations of Δ8 Complementation Constructs**

**ROP35SEQR6XRT AGTCAACGGAGGGCGTCACTC 5' RP for validation of 5' flank integration of Δ35 Complementation Construct**

**OROP18XF1A base CGCGGAAGTAACTCGAGTCGATG 5' FP for validation of integration of full length ROP18 constructs**

**OROP18XR1A base TTCTGTGTGGAGATGTTCCTGCTGTTC 3' RP for validation of integration of full length ROP18 constructs**

**OROP35XF1A base GTGCATGCATTCAAGCAGTTGTGTC 5' FP for validation of integration of full length ROP35 construct**

**OROP35XR1A base TTCGTTTTCCTGTTCATGGTCTTGTTCC 3' RP for validation of integration of full length ROP35 construct**

*FP indicates forward primer and RP indicates reverse primer.
